# Influence of sintering temperature on structural and optical properties of Cd_0.5_Cu_0.5_Cr_x_Fe_2−x_O_4_ ferrites

**DOI:** 10.1038/s41598-023-41214-1

**Published:** 2023-09-19

**Authors:** R. E. El shater, A. W. Awad, E. K. Abdel-Khalek, H. H. El-Bahnasawy, T. M. Meaz, Ehab A. Okba

**Affiliations:** 1 Physics Department, Faculty of Science, Tanta University, Tanta, 31527 Egypt; 2 Physics Department, Faculty of Science, Al-Azhar University, Nasr City, Cairo 11884 Egypt; 3 Chemistry Department, Faculty of Science, Tanta University, Tanta, 31527 Egypt

**Keywords:** Materials for optics, Physics

## Abstract

Two ferrite series were synthesized. One series has nanosize samples that have been prepared by the co-precipitation method, and the second series has the corresponding bulk samples that have been sintered at 1000 °C for 6 h. X-ray diffraction has been used to estimate the cubic spinel structure of both series. The crystallite size, theoretical density, and porosity of the nanomaterials are larger than those of the bulk materials. HRTEM analysis demonstrated the aggregation of nanoscale samples, including an average particle size of 9–22.5 nm. However, bulk specimens have a limited surface area. The agglomeration of the nanoparticles was seen in TEM images, in which the mean particle size was within the limit of the crystallite size (R) result and ranged from 14 to 20 nm. The appearance of the spinel phase in the samples was validated through Raman spectroscopy. Different cation occupation ratios in either tetrahedral or octahedral sites have been identified to be associated with an observable systematic shift and asymmetric flattening in Raman spectra with a variation in Cr^3+^ concentration. The optical characterization was performed using the UV/Vis methodology, and the results reveal that the absorption cutoff frequency declines as the chromium content rises. It was also estimated that the optical bandgap averaged 3.6 eV for nanosamples and 4.6 eV for overall bulk materials. The highest photoluminescence emission was seen at wavelengths between λ_em_ = 415 and 460 nm. The photoluminescence emission peaks of both bulk and nanoscale materials were red-shifted. These results accurately reflect the corresponding energy gap values for almost the same ranges. Sintering leads to a rise in photoluminescence.

## Introduction

Spinel structures are a group of compounds that are isotypes of crystalline spinel (MFe2O4), where M is a divalent cation. Due to their usefulness as magnetic materials^1^, semiconductors^2^, catalysts^3^, pigments, and protective coatings^4^. spinel products are widely exploited in the industry. The crystalline structure of spinel, which corresponds to the space group Fd3m, is a face-centered cubic (FCC) lattice with eight fundamental units in the cubic unit cell ^4^, Due to their significantly outstanding and distinctive properties compared to bulk materials and the corresponding nanoscale size of their crystal surfaces, nano-ferrites have become extensively deployed^5^. Magnetic interaction and cation distribution in sub-lattices determine the optical properties of magnetic spinel ferrites^6^. Optical materials are substances which are used to manipulate the inflow of light. This can include reflecting, absorbing, focusing or dividing an optical ray. The effectiveness of a specific material at each task is dynamically wavelength dependent; therefore, a full understanding of the intercourse between light and matter is vital. Optical Materials with novel and controlled electronic properties have wide applications, including computers, lighting, detectors, drug, and sustainability. Research in electronic and optical materials includes processing techniques for obtaining materials with controlled compositions and structures, characterization, and applications of these materials^7^. Phase change materials (PCMs) undergo rapid and drastic changes of their optical properties upon switching from one crystallographic phase to another one^8^.

Researchers have discovered and designed a variety of materials as a result of the demand for various devices based on intriguing electronic and optoelectronic characteristics. Since these materials' interactions with light and matter are significantly distinct from those of conventional materials, the nanoscale dimension alterations made them suitable for applications in optics^9^. Most materials' characteristics are dimensionally sensitive and respond in fundamentally distinct ways to grain size reduction. The confinement effect, which results from compact size, offers fascinating properties in nanomaterials that are not present in bulk, leading to multiple applications in numerous disciplines. Colloidal PbS quantum dots that show a strong quantum confinement effect have potential towards photovoltaic application. Promising constant photoluminescence (PL) peak energies of PbS and PbS/MnS core shell could be advantageous particularly for biological marker application. The effect of laser light on the photoluminescence of quantum dots can be considered as a useful standard for selection of appropriate nanocrystals for certain applications^10^. Nanostructured spinel metal oxides are well-known for their distinct characteristics that are critical to both scientific and technological progress. The study of optical nonlinearities has led to the development of both normal and inverse spinel ferrites, which are used in optical data storage, optical communications, data processing, and optical computing^11,12^. Moreover, there hasn't been much extensive research on spinel-structured materials' optically limiting applications up until now^6^.

Spinel structures come into two main categories: normal and inverse. Numerous magnetic materials use spinel ferrites^13^. Ferrite contains copper (CuFe_2_O_4_), which is inverse spinel^4^ and identified by the existence of ferric ions in the tetrahedral (A) sites and ferric and copper ions in the octahedral (B) sites, as can be seen by the formula ${{[{({\mathrm{Fe}}_{ }}^{3+}]}_{ }}^{\mathrm{T}}{{{({\mathrm{Cu}}^{2+}{\mathrm{Fe}}_{ }}^{3+})}^{\mathrm{O}}}_{ }{\mathrm{O}}_{4}^{2-}$. A minor fraction of Cu^2+^ ions in doped copper ferrite could potentially migrate from the octahedral B-site to the tetrahedral one^13^. The proportion of copper ions at B-sites refers to the degree of inversion, or the inversion factor, as can be seen by expression $({\mathrm{Cu}}_{1-\updelta }{\mathrm{Fe}}_{\updelta }) [{\mathrm{Cu}}_{\updelta }{\mathrm{Fe}}_{2-\updelta }]{\mathrm{O}}_{4}$, where is a numeric value between 0 and 1, where even the values 0 and 1 denote, alternately, the inverse and normal occurrences. According to the formula, cadmium ferrite (CdFe_2_O_4_) is a normal form of spinel in which ferric ions engage the octahedral (B) sites and cadmium ions inhabit the tetrahedral (A) sites. ${{[{\mathrm{M}}^{2+}]}_{ }}^{\mathrm{T}}{{{({\mathrm{Fe}}_{2 }}^{3+})}^{\mathrm{O}}}_{ }{{\mathrm{O}}^{2-}}_{4}$. Higher Cr^3+^ (x > 0.02) concentrations exhibit a propensity alone for populating the B-site while somehow destabilizing the proportion of ferric ions in both the A and B sites^1^. The spinel ferrite methodology and sintering temperatures already have the biggest effects on the aforementioned features.

Several methods have been employed to synthesize nano-Cr ferrite with controlled particle size using chemical and physical routes, including co-precipitation^14^, citrate precursor method^13^, sol–gel method^5^, combustion method^6^*,* hydrothermal^15^, and solvothermal^16^. UV-visible spectroscopy is a useful tool for investigating the relationship between band gap and crystallite size. Nanoparticles' electronic and optical properties are determined by their size and shape^6^. Metal nanoparticles have primarily been studied due to their distinctive optical properties^17,18^. Surface plasmon resonance (SPR) is highly dependent on the nanomaterial’s size, shape, and nature. Indeed, tuning those parameters enables a controllable wavelength range between the ultraviolet (UV) and near-infrared (NIR). Photoluminescence (PL) is one of the more interesting applications. The most powerful Photoluminescence spectra are created by direct recombination of photo-generated holes formed in the tetrahedral and octahedral sites of the crystals, oxygen vacancies trapping electrons, and the electronic transitions in Fe^+3^ ions from the 3d^5^ state to the 3d^4^ 4 s state^19^.

If nanoparticles (NPs) are diffused, they are quasi-spherical particles. The quasi-spherical particles show a bimodal size distribution, where UV-Vis absorption spectra and the surface plasmon resonance SPR of multiple peaks are observed. Due to alterations in surface polarisation, the shape and size of metallic NPs influence their spectral properties. Visible to infrared regions exhibit SPR absorption from various shapes, including spheres, triangles, cubes, prisms, bipyramids, octahedrons, nanorods, nanoshells, and nanostars. Due to an increase in charge separation, when the boundaries or sharpness of an NP become sharper, the extinction spectra shift to the red, and when an NP’s symmetry improves, the SPR intensity increases^20^. Non-spherical NPs tend to have multiple, red-shifted peaks, whereas spherical nanorods can be polarised along two axis. The shape of the particles is the primary response to how each field enhancement is created.

The variety of geometrical parameters influences their spectral features, for example, by raising the aspect ratio of nanorods (NR). Because of the anisotropy, the dipolar mode splits into two modes (transversal and longitudinal) with different refractometric sensitivities, absorption intensities, and wavelength positions^21^. Multipolar resonant frequencies can appear when many complex nanoparticle particles are used. Another important mechanism involves the collective interactions of NPs, which change the optical response of the whole NPS assembly. This coupling allows for the creation of a new plasmonic tuning mechanism that involves varying the interparticle distances or periods in arrayed nanostructures. This is critical for NP arrays and self-assembled nanostructures' spectral behavior^22^. Changing the size of the nanospheres (NS) results in different optical properties for each nanostructure. Furthermore, the way interparticle coupling occurs among these nanomaterials is strongly dependent on the anisotropy of the NPS and, thus, nanostructures.

The goal of this study is to make and measure how the doping ratio affects the structural, morphological, and optical properties of both smelted and newly made spinel ferrites. The major emphasis was on the optical characteristics of the nano-ferrites under study, and the present endeavor aims to maximize these characteristics.

## Experimental method

### Materials and method

Using the chemical co-precipitation method, nanocrystalline chromium-doped copper-cadmium ferrites with the stoichiometric formula ${\mathrm{Cd}}_{0.5}{\mathrm{Cu}}_{0.5}{\mathrm{Cr}}_{\mathrm{x}}{\mathrm{Fe}}_{2-\mathrm{x}}{\mathrm{O}}_{4}$ with steps x = 0, 0.05, 0.10, 0.20, 0.40, and 0.80 were made. Analytical grade cadmium nitrate $(\mathrm{Cd}{\left({\mathrm{NO}}_{3}\right)}_{2}.4{\mathrm{H}}_{2}\mathrm{O})$, cupper nitrate $\left[\mathrm{Cu}{\left({\mathrm{NO}}_{3}\right)}_{2}.4{\mathrm{H}}_{2}\mathrm{O}\right]$, chromium nitrate $[\mathrm{Cr}{\left({\mathrm{NO}}_{3}\right)}_{3}. 9{\mathrm{H}}_{2}\mathrm{O}]$ and iron nitrate $[\mathrm{Fe}{\left({\mathrm{NO}}_{3}\right)}_{3}.9{\mathrm{H}}_{2}\mathrm{O}]$ as starting materials, were mixed initially at a molar ratio of 1:2 M. The divalent metal molarity was 0.2 M, and the trivalent metal molarity was 0.4 M. The metal nitrates were dissolved in distilled water using a magnetic stirrer in 125 mL for all samples, and 3M of sodium hydroxide (NaOH) was dissolved in the mixture. For 2 h, the solution was heated to 90 °C until precipitation occurred. The precipitations were washed several times in deionized water to remove undesired salt residuals before being dried in an oven at 100 °C overnight and ground to a fine powder in an agate mortar^23^. Considerable amounts of the as-prepared samples were calcined for 12 h at a high temperature of 1000 °C with a temperature step rate of 3 degree per minute, to study the effect of annealing on the structure, optical, and other physical properties of the samples. In the following Table 1, the names of the prepared samples are listed. The as-prepared samples have grain sizes on nanoscale, which are named nano samples, while the sintered samples have grain sizes on microscale, which are named bulk samples.

**Table 1:** Symbols of the as-prepared and bulk samples.

Sample	Nano-samples	Bulk samples
Cd_0.5_Cu_0.5_Cr_0.00_Fe_2_O_4_	Cr00N	Cr00B
Cd_0.5_Cu_0.5_Cr_0.05_Fe_1.95_O_4_	Cr05N	Cr05B
Cd_0.5_Cu_0.5_Cr_0.10_Fe_1.9_O_4_	Cr10N	Cr10B
Cd_0.5_Cu_0.5_Cr_0.20_Fe_1.8_O_4_	Cr20N	Cr20B
Cd_0.5_Cu_0.5_Cr_0.40_Fe_1.6_O_4_	Cr40N	Cr40B
Cd_0.5_Cu_0.5_Cr_0.80_Fe_1.2_O_4_	Cr80N	Cr80B

### Characterization techniques

X-ray Diffraction (XRD), model PANalytical (X'pert Pro, Netherlands), furnished with a high-intensity Cu k radiation source ($\uplambda =0.154\mathrm{ nm}$, 40 mA, 40 kV) in the 2θ range 10°–80°, has been used to characterize the crystalline structure of the synthesized samples.

Surface morphology was measured using field emission scanning electron microscopy (FESEM) images and energy dispersive X-ray spectra (EDX), which were taken with a Hitachi S-4800 scanning electron microscope. Assessment of morphology has been achieved using SEM. Chemical analysis has made use of EDX.

To examine the particle distribution, high resolution transmission electron microscope analysis (HRTEM) was employed. HRTEM assessment was carried out on a 200 kV-operated JEOL- JEM-2100. The modifications in the cation distribution have been substantiated by RAMAN analysis. An Ar green laser has been used to record the Raman spectra using a JASCO NRS-1000 micro-spectrometer with an excitation source of 514.5 nm.

Diffuse Reflectance Spectrophotometry (DRS), a form of UV spectrophotometer, is developed to evaluate the linear optical features and estimate the band gap energies. Jasco model V-570 scanning spectrometer used for DRS. The mechanism of recombination and the relative energy position of sub-band-gap defect states on the surface of metal oxides have been researched employing photoluminescence emission spectroscopy (PL). A fluorimetric study was conducted using a Japanese-made spectrofluorometer, the Jasco FP-6500, for both excitation and emission spectra.

## Result and discussion

### X-ray diffraction analysis

**Figure 1:**
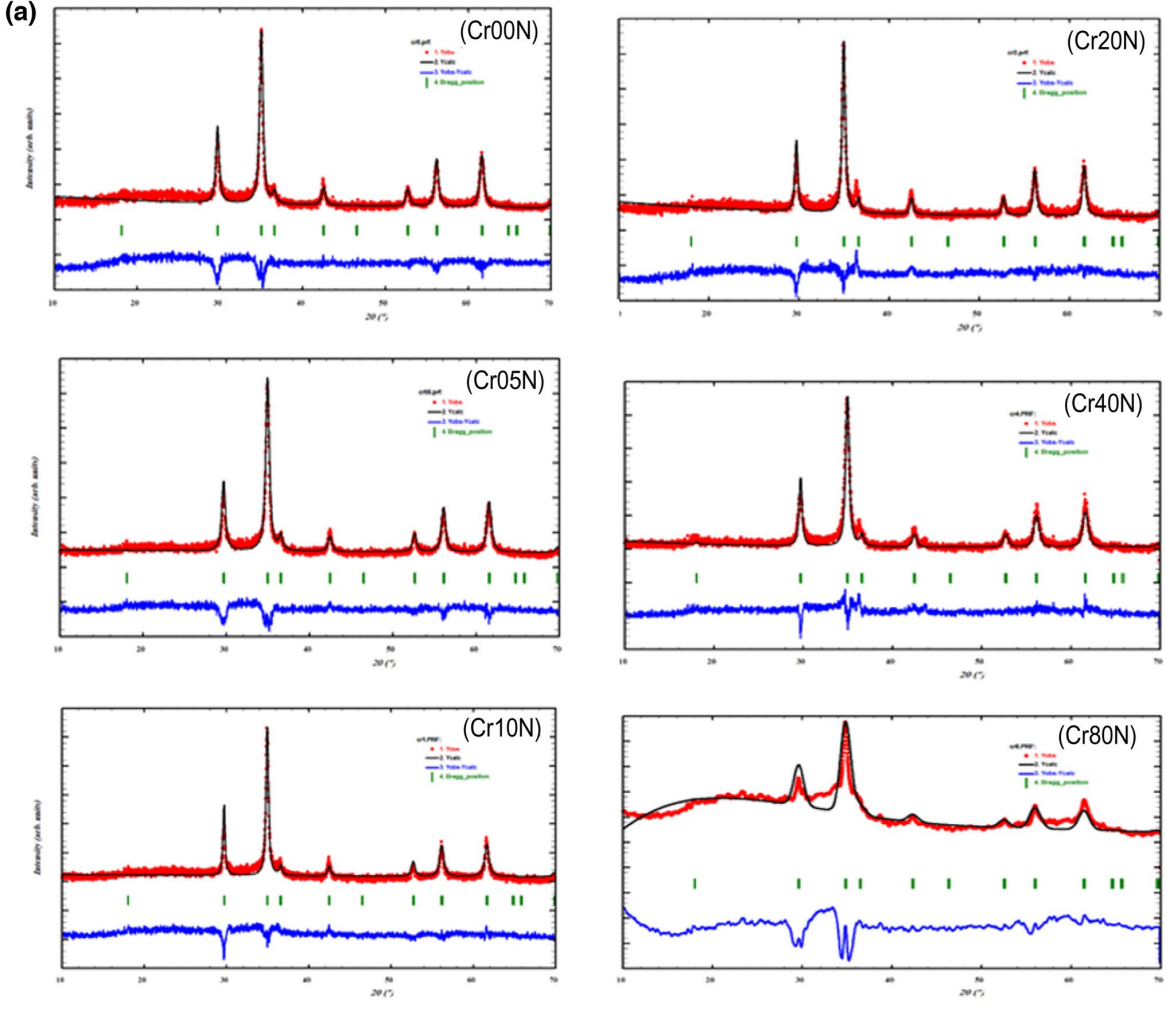

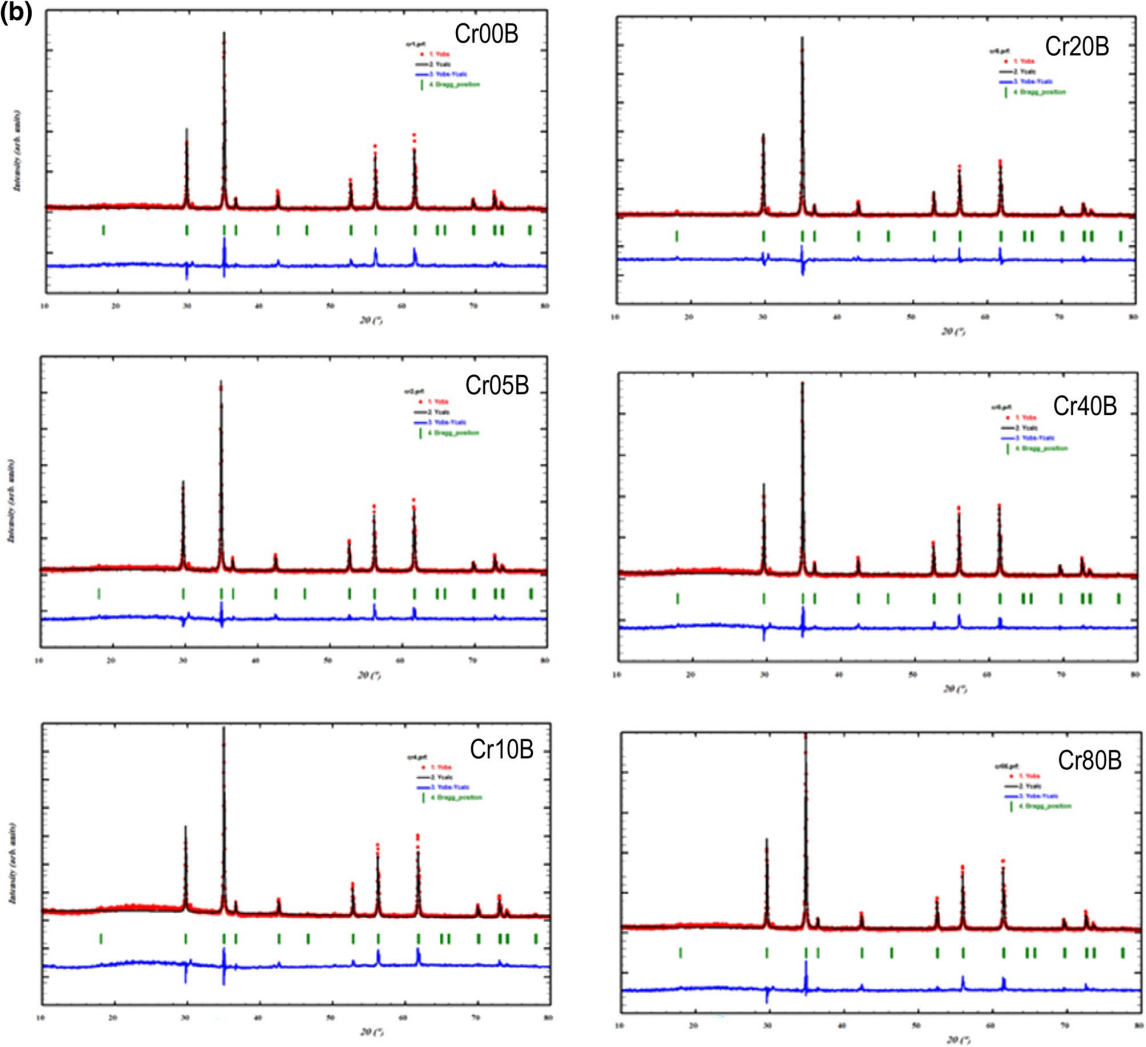
(**a**): The XRD pattern and the Rietveld analysis of the as prepared samples. (**b**): the XRD pattern and the Rietveld analysis of the bulk samples.

X-ray diffraction (XRD) was used to investigate the structural of the prepared samples, as shown in Fig. 1a. The most intense peaks in the nano ferrite samples were indexed as (220), (311), (222), (400), (422), (511), (440), and (533) were confirmed to be well matched with the single-phase cubic spinel structure [compared with JCPDS card No. 00–022-1086]. For the sintered samples, the XRD patterns showed sharp lines corresponding to cubic spinel single-phase structure, as shown in Fig. 1b that confirmed to be matched with the single-phase cubic spinel structure [compared with JCPDS card No. 00–001-1121]. The XRD pattern of the sintered samples revealed that the width of the peaks had narrowed and became more intense as a result of the structures' elimination of common sources of strain can be given as dislocations, stacking faults, long-range internal stresses, grain boundaries, chemical heterogeneities and point defects^24,25^.

The peak (311) is almost the spinel ferrites’ main distinctive peak^26^. According to Bragg’s relation, the inter-planar distance (d) is related to the cubic structure's lattice constant (${a}_{exp}$) as follows:

1 $${a}_{exp}={\mathbf{d}({\mathbf{h}}^{2}+{\mathbf{k}}^{2}+{\mathbf{l}}^{2})}^{{1}/{2}}$$

where $h, k and l$ are the miller indices of the planes associated with the characteristic peak*.* The lattice parameters (a_exp_) for both nano and bulk series in Tables 2 and 3 were calculated using the Rietveld analysis, that decrease with increasing Cr^3+^ ions substitution in the system. The decrease in the lattice parameter was attributed to the replacement of larger Fe^3+^ (0.64) ion by smaller Cr^3+^ (0.61 Å) ions*.* Furthermore, the size of crystallite and lattice strain influences X-ray diffraction patterns. Accordingly; Williamson-Hall analysis (W–H) is being used to separate the crystallite size and strain-induced deformation peak by taking the peak width broadening as a function of 2θ into account^27^. The total broadening $(\upbeta )$ of the peak is the result of the combined broadening due to crystallites size (${\upbeta }_{\mathrm{D}})$ and broadening due to lattice strain (β_ε_). As a result, total broadening is obtained by:

**Table 2:** The calculated XRD parameters of the as-prepared sample: d-spacing, lattice constant (${\mathbf{a}}_{\mathbf{e}\mathbf{x}\mathbf{p}})$, crystallite size (D), Molecular Wight (M_w_), x-ray density (${\rho }_{x}$), measured density (${\rho }_{m}$), porosity (P), and strain (ε).

Sample	d-(311)	${\mathbf{a}}_{\mathbf{e}\mathbf{x}\mathbf{p}}$	D	Mw	${{\varvec{\rho}}}_{\mathbf{X}}$	${{\varvec{\rho}}}_{{\varvec{m}}}$	$\mathbf{P}$	ε	δ
	Ǻ	Ǻ	nm	**g/mol**	$\mathbf{g}/{\mathbf{c}\mathbf{m}}^{3}$	$\mathbf{g}/{\mathbf{c}\mathbf{m}}^{3}$	$(\boldsymbol{\%})$	$(\boldsymbol{\%})$	**nm**^**−2**^
Cr00N	2.56481	8.5065	18.247	263.668	5.6924	2.5610	55	6.33 × 10^–3^	3.00 × 10^–3^
Cr05N	2.56858	8.5190	16.848	263.475	5.6633	2.3620	58	6.86 × 10^–3^	3.52 × 10^–3^
Cr10N	2.56648	8.5120	25.288	263.283	5.6731	2.5061	56	4.57 × 10^–3^	1.56 × 10^–3^
Cr20N	2.56606	8.5107	22.529	262.898	5.6674	2.3243	59	5.13 × 10^–3^	1.97 × 10^–3^
Cr40N	2.56713	8.5142	17.525	262.128	5.6438	2.2512	60	6.59 × 10^–3^	3.26 × 10^–3^
Cr80N	2.57488	8.5398	6.105	260.588	5.5604	2.2312	60	1.90 × 10^–2^	2.68 × 10^–2^

**Table 3:** The calculated XRD parameters of the bulk sample: d- spacing (d), lattice constant $({\mathrm{a}}_{\mathrm{exp}}$), crystallite size (D), x-ray density (${\rho }_{x}$), measured density (${\rho }_{m}$), porosity (P), strain (ε), and dislocation (δ).

Sample	$\mathbf{d}$	${\mathrm{a}}_{\mathrm{exp}}$	D	${\rho }_{\mathrm{X}}$	${\rho }_{m}$	**P**	**Strain**	**δ**
	Ǻ	Ǻ	nm	$\mathrm{g}/{\mathrm{cm}}^{3}$	$\mathrm{g}/{\mathrm{cm}}^{3}$	$(\%)$	$(\%)$	nm^−2^
Cr00B	2.57463	8.5391	92.39	5.627	4.2252	25	1.25 × 10^–3^	1.17 × 10^–4^
Cr05B	2.57362	8.5357	88.09	5.630	4.0841	27	1.31 × 10^–3^	1.29 × 10^–4^
Cr10B	2.57156	8.5289	91.05	5.639	4.0145	29	1.27 × 10^–3^	1.21 × 10^–4^
Cr20B	2.56638	8.5117	77.07	5.665	4.212	26	1.50 × 10^–3^	1.68 × 10^–4^
Cr40B	2.56069	8.4928	96.77	5.687	4.263	25	1.19 × 10^–3^	1.07 × 10^–4^
Cr80B	2.56018	8.4898	71.30	5.659	4.397	22	1.62 × 10^–3^	1.97 × 10^–4^

2 $${\varvec{\upbeta}}={{\varvec{\upbeta}}}_{\mathbf{D}}+{{\varvec{\upbeta}}}_{{\varvec{\upvarepsilon}}}=\frac{\mathbf{k}{\varvec{\uplambda}}}{\mathbf{D}\mathbf{C}\mathbf{o}\mathbf{s}({\varvec{\uptheta}})}+\frac{4{\varvec{\upvarepsilon}}\mathbf{S}\mathbf{i}\mathbf{n}({\varvec{\uptheta}})}{\mathbf{C}\mathbf{o}\mathbf{s}({\varvec{\uptheta}})}$$

where β is the radiant peak's full width at half maximum (FWHM) that was obtained from Rietveld analysis, $\mathrm{k}$ is the shape factor (k = 0.9), λ is the X-ray wavelength ($1.54056$ Å), $\uptheta$ is the diffraction angle, D is the crystallite size (nm) and ε is the micro strain. The broadening ${\upbeta }_{\mathrm{hkl}}$ (FWHM) and 2θ (position of peak center in degrees) were calculated by fitting the diffraction peak profile to a Gaussian function. Rearranging Eq. (2) gives:

3 $${\varvec{\upbeta}}\mathbf{C}\mathbf{o}\mathbf{s}\left({\varvec{\uptheta}}\right)=\frac{\mathbf{k}{\varvec{\uplambda}}}{\mathbf{D}}+4{\varvec{\upvarepsilon}}\mathbf{S}\mathbf{i}\mathbf{n}\left({\varvec{\uptheta}}\right)$$

**Figure 2:**
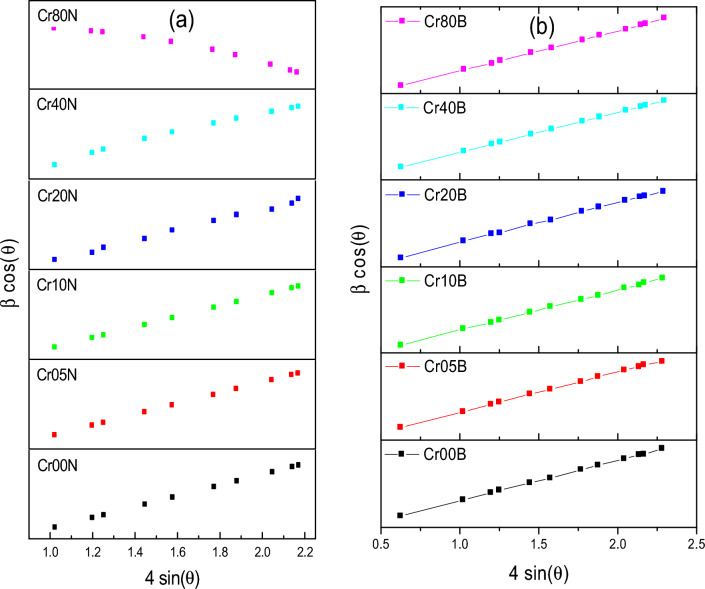
The W–H plot of (**a**) the as-prepared samples and (**b**) the bulk samples.

The relation between $\mathrm{\beta cos}(\uptheta$) and 4sin(θ) was fitted to a straight line as shown in Fig. 2a,b. The slope of this straight line shows the intrinsic strain and the point where the line meets the Y-axis shows the average particle size of the nanoferrite and sintered bulk ferrite as they are. The average crystallite size and strain can be inferred from the W–H plot in Fig. 2b using Y-intercept extrapolation and the slope of the line, i.e., $\mathrm{D}=\frac{\mathrm{K\lambda }}{\mathrm{Y\,intercept}}$ and ε = slop. The average crystallite size for nano-ferrites was found to be in the range of (25–6 nm) and for bulk ferrite in the range of (96–71 nm), as reported in Tables 2 and 3, respectively. By increasing Cr^3+^ ion content, the value of crystallite size decreased trendy for both nano and bulk series. This is due to the release of latent heat at the surface, which elevated the local temperature, slowed the development process, and reduced ferrite concentration in the vicinity^28^. The variance of ε demonstrates the mechanical properties of the materials. Its positive number indicates tensile tensions^29^. The values of dislocations (δ) for the as prepared materials in the order 10^–3^ while Values of dislocations for the bulk materials in the order of 10^–4^ which reveal improving and completing the crystallization of the bulk crystals^30,31^. The decrease of δ may point to transition process from nanograins structure into microstructure samples.

$$\delta =\frac{1}{{D}^{2}}$$

The measured densities ${\rho }_{m}$ were accurately determined using geometrical methods in Eq. (4). This was accomplished by determining the mass, diameter, and thickness of each pellet. The X-ray densities ${\rho }_{x}$ were calculated from the lattice parameter values using the formula (5);

4 $${{\varvec{\rho}}}_{{\varvec{m}}}=\frac{{\varvec{m}}}{{\varvec{\pi}}{{\varvec{r}}}^{2}{\varvec{t}}}$$

5 $${{\varvec{\rho}}}_{{\varvec{x}}}=\frac{{\varvec{Z}}{\varvec{M}}}{{{\varvec{N}}}_{{\varvec{A}}}{{\varvec{a}}}^{3}}$$

where Z is the number of molecules per unit cell (for spinel ferrites, Z = 8), M is the molecular weight of the sample (g/mol), N_A_ is the Avogadro's number (6.023 × 10^23^ atom/mol), and m, r, and t are the mass, radius, and thickness, respectively, of each pellet. Both bulk and X-ray density data increase with Cr substitution. Each pellet's porosity (P) was calculated using the following formula (6):

6 $${\varvec{P}}=\frac{{{\varvec{\rho}}}_{{\varvec{x}}}-{{\varvec{\rho}}}_{{\varvec{m}}}}{{{\varvec{\rho}}}_{{\varvec{x}}}}$$

As evaluated in Tables 2 and 3 X-ray densities of bulk densities are greater than nan-sample densities, while the porosity of bulk densities are lower than nan-sample densities. This is as a result of the sintering process eliminating the flaws and empty spaces (voids) in the nanoparticles. Samples with high densities appear to be less porous. The density of polycrystalline ferrites plays a significant role in controlling their various properties. It is observed that the X-ray density (${{\varvec{\rho}}}_{{\varvec{x}}}$) of each sample is greater than the corresponding measured density (${{\varvec{\rho}}}_{{\varvec{m}}}$). This may be due to some unavoidable pores created during the sintering process.

Spinel oxide's crystal structure is described by three crystallographic parameters: the lattice constant (${\mathrm{a}}_{\mathrm{th}}$), the oxygen position parameter ($u$), and the distribution of cations in the A and B sites. The ionic radius and the lattice parameter are related; the increase in the lattice parameter is proportional to the rise in the ionic radius. Using the cation distribution and the following equations, the theoretical lattice constant (${\mathrm{a}}_{\mathrm{th}}$), the radius of the ions at the octahedral site (${\mathrm{r}}_{\mathrm{b}}$), and the radius of the ions at the tetrahedral site (${\mathrm{r}}_{\mathrm{a}}$) for spinel systems have been estimated:

7 $${{\varvec{a}}}_{{\varvec{t}}{\varvec{h}}}=\frac{8}{3\sqrt{3}}[\left({{\varvec{r}}}_{{\varvec{a}}}+{{\varvec{R}}}_{0}\right)+\sqrt{3}\left({{\varvec{r}}}_{{\varvec{b}}}+{{\varvec{R}}}_{0}\right)]$$

8 $${{\varvec{r}}}_{{\varvec{a}}}={\mathbf{a}}_{{{\mathbf{C}\mathbf{d}}_{\mathbf{T}}}^{2+}}\boldsymbol{*}{\mathbf{r}}_{{{\mathbf{C}\mathbf{d}}_{\mathbf{T}}}^{2+}}+{\mathbf{a}}_{{{\mathbf{C}\mathbf{u}}_{\mathbf{T}}}^{2+}}\boldsymbol{*}{\mathbf{r}}_{{{\mathbf{C}\mathbf{u}}_{\mathbf{T}}}^{2+}}+{\mathbf{a}}_{{{\mathbf{F}\mathbf{e}}_{\mathbf{T}}}^{3+}}\boldsymbol{*}{\mathbf{r}}_{{{\mathbf{F}\mathbf{e}}_{\mathbf{T}}}^{3+}}$$

9 $${{\varvec{r}}}_{{\varvec{b}}}=\frac{1}{2}({\mathbf{a}}_{{{\mathbf{C}\mathbf{u}}_{\mathbf{O}}}^{2+}}{\mathbf{*}\mathbf{r}}_{{{\mathbf{C}\mathbf{u}}_{\mathbf{O}}}^{2+}}+{\mathbf{a}}_{{{\mathbf{C}\mathbf{r}}_{\mathbf{O}}}^{3+}}{\mathbf{*}\mathbf{r}}_{{{\mathbf{C}\mathbf{r}}_{\mathbf{O}}}^{3+}}+{\mathbf{a}}_{{{\mathbf{F}\mathbf{e}}_{\mathbf{O}}}^{3+}}\boldsymbol{*}{\mathbf{r}}_{{{\mathbf{F}\mathbf{e}}_{\mathbf{O}}}^{3+}})$$

where, ${\mathrm{R}}_{0}$ is the radius of the oxygen ion (1.32 Å) and ${r}_{a}$ and ${r}_{b}$ are the ionic radii of tetrahedral (A-site) and octahedral (B-site), respectively^32^. According to Shannon; ${\mathrm{r}}_{{{\mathrm{Cd}}_{\mathrm{T}}}^{2+}}$(0.95 A), ${\mathrm{r}}_{{{\mathrm{Cu}}_{\mathrm{T}}}^{2+}}$(0.73 A), ${\mathrm{r}}_{{{\mathrm{Fe}}_{\mathrm{T}}}^{3+}}$(0.55 A) are the ionic radii of Cd^2+^, Cu^2+^ and Fe^3+^ ions in the tetrahedral sites respectively. ${\mathrm{r}}_{{{\mathrm{Cu}}_{\mathrm{O}}}^{2+}}$(0.73 A), ${\mathrm{r}}_{{{\mathrm{Cr}}_{\mathrm{O}}}^{3+}}$(0.615 A), ${\mathrm{r}}_{{{\mathrm{Fe}}_{\mathrm{O}}}^{3+}}$(0.64 A) are the ionic radii of Cu^2+^, Cr^3+^ and Fe^3+^ ions in the octahedral sites respectively ^33^. The values of ${r}_{a}$ and ${r}_{b}$ will be heavily influenced by the system's cation distribution. In order to calculate ${r}_{a}$ and ${r}_{b}$, the general cation distribution Eq. (10) is suggested for the composition ${\mathrm{Cd}}_{0.5}{\mathrm{Cu}}_{0.5}{\mathrm{Cr}}_{\mathrm{x}}{\mathrm{Fe}}_{2-\mathrm{x}}{\mathrm{O}}_{4}$:

10 $${[{\mathbf{C}\mathbf{d}}_{0.5}{\mathbf{C}\mathbf{u}}_{0.5-{\varvec{\updelta}}}{\mathbf{F}\mathbf{e}}_{{\varvec{\updelta}}}]}_{\mathbf{A}}{[{\mathbf{C}\mathbf{u}}_{{\varvec{\updelta}}}{\mathbf{F}\mathbf{e}}_{2-\mathbf{x}-{\varvec{\updelta}}}{\mathbf{C}\mathbf{r}}_{\mathbf{x}}]}_{\mathbf{B}}{\mathbf{O}}_{4}$$

Chromium ions are known to occupy B-sites in the spinel lattice, which makes the A sites spread out in a very different way^1^. The lattice parameter is shown to decrease as the Cr content increases. The lattice constant values are within the predicted range for spinel cubic ferrites. Hypotheses were made about the cation distributions at A- and B-sites to explain why lattice constants change, and lattice constants were calculated as shown in Tables 4 and 5. To get the expected lattice constant to agree with the lattice constant determined from X-ray diffraction peaks, the cation distribution in the spinel lattice was adjusted. In this instance, the distribution of cations has been presumptively inferred. According to Shannon^33^ It can be seen that ${\mathrm{r}}_{\mathrm{A}}$ and ${\mathrm{r}}_{\mathrm{B}}$ decrease with increasing Cr^3+^ concentration. This is due to the fact that in the tetrahedral site, Cu^2+^ ions (0.73 Å) with a larger ionic radius are replaced by low spin Fe^3+^ ions (0.55 A) with a smaller ionic radius. Some high spin Fe^3+^ ions (0.49 Å) also migrate to (A) sites as a result of the substitution process. However, at (B) sites, high-spin Fe^3+^ ions with a smaller ionic radius are substituted by Cu^2+^ ions with a higher ionic radius and Cr^3+^ ions with an equivalent ionic radius for low-spin Fe^3+^ ions. Qi et al.^34^ have created a model to link the size and shape dependent lattice parameters of nanoparticles. The particle shape difference has been taken into account in this model by introducing a shape factor^27^. Furthermore, as shown in Table 2, it was predicted that the lattice parameters of nanoparticles decrease as crystallite size decreases.

**Table 4:** The suggested cation distribution of as-prepared samples.

Sample	Tetrahedral-site	Octahedral-site	${\mathbf{a}}_{\mathbf{t}\mathbf{h}}$
			(Ǻ)
Cr00N	$({\mathrm{Cd}}_{0.5}{\mathrm{Cu}}_{0.273}{\mathrm{Fe}}_{0.227}{)}_{\mathrm{T}}$	${({\mathrm{Cu}}_{0.227}{\mathrm{Fe}}_{1.773})}_{\mathrm{O}}$	8.5165
Cr05N	$({\mathrm{Cd}}_{0.5}{\mathrm{Cu}}_{0.222}{\mathrm{Fe}}_{0.278}{)}_{\mathrm{T}}$	${({\mathrm{Cu}}_{0.278}{\mathrm{Cr}}_{0.05}{\mathrm{Fe}}_{1.672})}_{\mathrm{O}}$	8.50686
Cr10N	$({\mathrm{Cd}}_{0.5}{\mathrm{Cu}}_{0.207}{\mathrm{Fe}}_{0.293}{)}_{\mathrm{T}}$	${({\mathrm{Cu}}_{0.293}{\mathrm{Cr}}_{0.1}{\mathrm{Fe}}_{1.607})}_{\mathrm{O}}$	8.50283
Cr20N	$({\mathrm{Cd}}_{0.5}{\mathrm{Cu}}_{0.204}{\mathrm{Fe}}_{0.296}{)}_{\mathrm{T}}$	${({\mathrm{Cu}}_{0.296}{\mathrm{Cr}}_{0.2}{\mathrm{Fe}}_{1.504})}_{\mathrm{O}}$	8.499
Cr40N	$({\mathrm{Cd}}_{0.5}{\mathrm{Cu}}_{0.202}{\mathrm{Fe}}_{0.298}{)}_{\mathrm{T}}$	${({\mathrm{Cu}}_{0.298}{\mathrm{Cr}}_{0.4}{\mathrm{Fe}}_{1.302})}_{\mathrm{O}}$	8.49205
Cr80N	$({\mathrm{Cd}}_{0.5}{\mathrm{Cu}}_{0.275}{\mathrm{Fe}}_{0.225}{)}_{\mathrm{T}}$	${({\mathrm{Cu}}_{0.225}{\mathrm{Cr}}_{0.8}{\mathrm{Fe}}_{0.975})}_{\mathrm{O}}$	8.49018

**Table 5:** The suggested cation distribution of the bulk samples.

Sample	Tetrahedral-site	Octahedral-site	${\mathbf{a}}_{\mathbf{t}\mathbf{h}}$
			(Ǻ)
Cr00B	$({\mathrm{Cd}}_{0.5}{\mathrm{Cu}}_{0.431}{\mathrm{Fe}}_{0.069}{)}_{\mathrm{T}}$	${({\mathrm{Cu}}_{0.069}{\mathrm{Fe}}_{1.931})}_{\mathrm{O}}$	8.5414
Cr05B	$({\mathrm{Cd}}_{0.5}{\mathrm{Cu}}_{0.312}{\mathrm{Fe}}_{0.188}{)}_{\mathrm{T}}$	${({\mathrm{Cu}}_{0.188}{\mathrm{Cr}}_{0.05}{\mathrm{Fe}}_{1.762})}_{\mathrm{O}}$	8.521
Cr10B	$({\mathrm{Cd}}_{0.5}{\mathrm{Cu}}_{0.276}{\mathrm{Fe}}_{0.224}{)}_{\mathrm{T}}$	${({\mathrm{Cu}}_{0.224}{\mathrm{Cr}}_{0.1}{\mathrm{Fe}}_{1.676})}_{\mathrm{O}}$	8.5137
Cr20B	$({\mathrm{Cd}}_{0.5}{\mathrm{Cu}}_{0.255}{\mathrm{Fe}}_{0.245}{)}_{\mathrm{T}}$	${({\mathrm{Cu}}_{0.245}{\mathrm{Cr}}_{0.2}{\mathrm{Fe}}_{1.555})}_{\mathrm{O}}$	8.50704
Cr40B	$({\mathrm{Cd}}_{0.5}{\mathrm{Cu}}_{0.134}{\mathrm{Fe}}_{0.366}{)}_{\mathrm{T}}$	${({\mathrm{Cu}}_{0.366}{\mathrm{Cr}}_{0.4}{\mathrm{Fe}}_{1.234})}_{\mathrm{O}}$	8.48136
Cr80B	$({\mathrm{Cd}}_{0.5}{\mathrm{Cu}}_{0.077}{\mathrm{Fe}}_{0.423}{)}_{\mathrm{T}}$	${({\mathrm{Cu}}_{0.423}{\mathrm{Cr}}_{0.8}{\mathrm{Fe}}_{0.777})}_{\mathrm{O}}$	8.45907

### Scanning electron microscope

**Figure 3:**
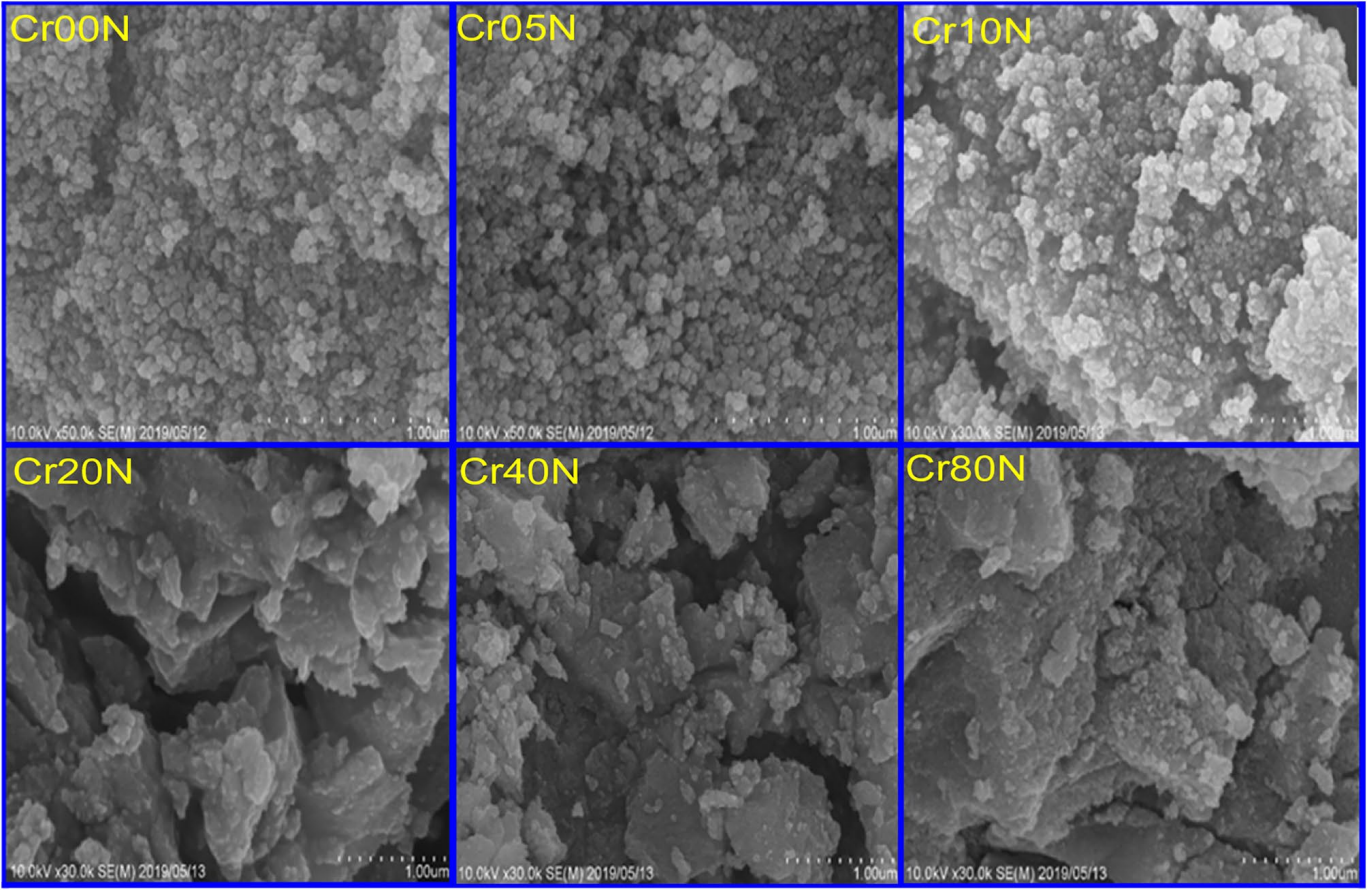
SEM images of the as-prepared samples.

**Figure 4:**
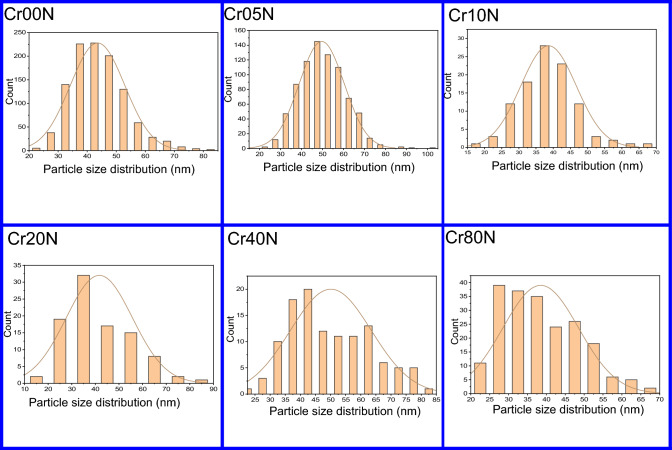
Particle size distribution of the as prepared samples.

**Figure 5:**
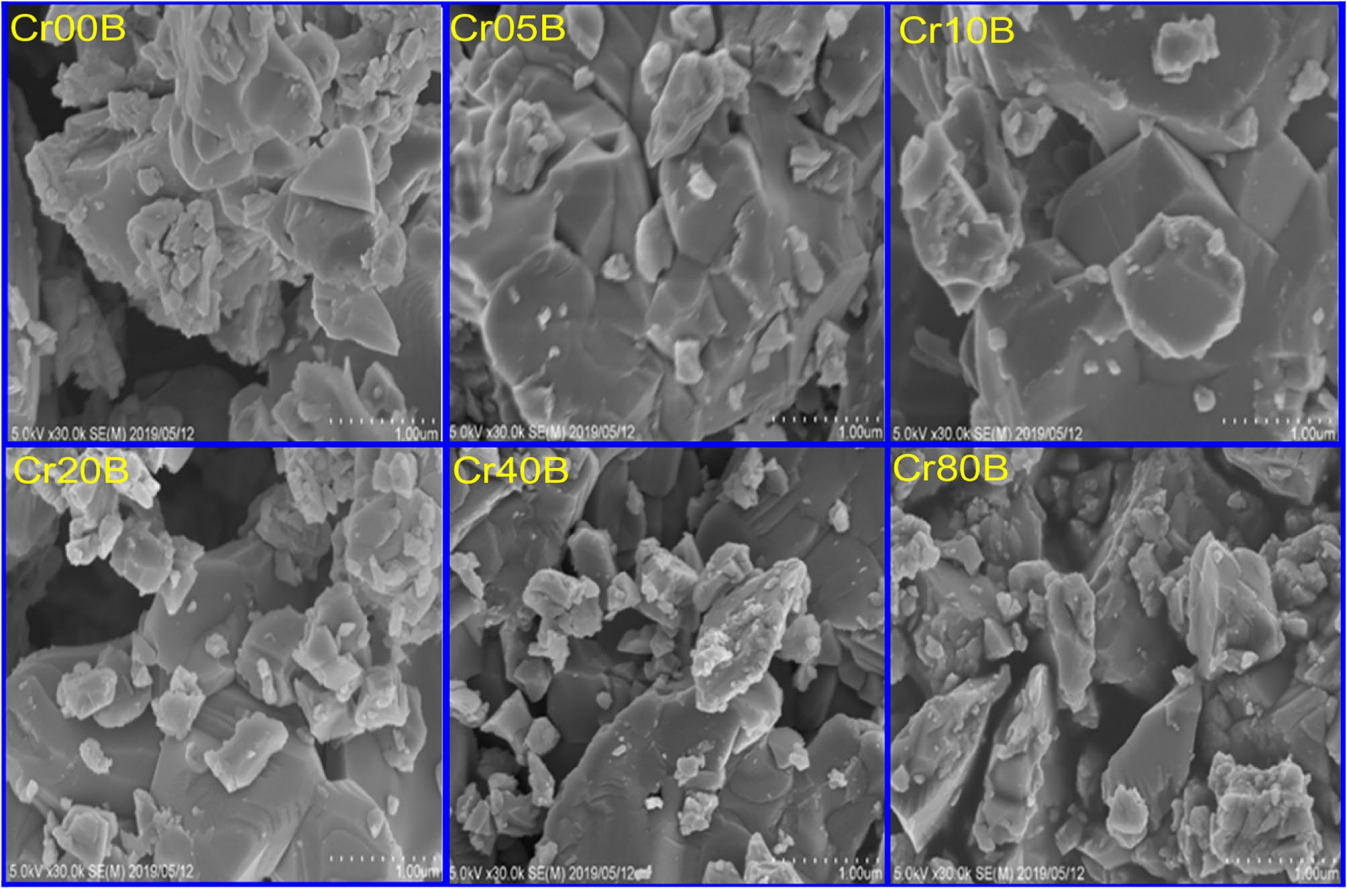
SEM images of the bulk samples.

The particle agglomeration phenomenon in Figs. 3 and 5 is allowed by the surface activity, which demonstrates that all the particles are thoroughly cemented. The magnetic interactions among particle surfaces may be the cause of the observed particle agglomeration^14^. An increase in doping ion concentration causes a rise in agglomeration and clustering, which may be induced by oxygen vacancies and porosity that restrict grain dispersion^35^. Figure 4 demonstrated that the average particle size of the nanoferrite series has values in the range of 40–50 nm. These values are higher than the crystallite size estimated from X-rays which has values in the range of 15–20 nm. The crystallite size of the samples determined by XRD is not always the same as the particle size determined by SEM images due to the existence of polycrystalline aggregates^27^. This difference can be attributed to the fact that the SEM image provides the particle size of the material. Taking into consideration that the particle is composed of an agglomeration of many grains, and every grain consists of many crystallites. The crystallite size of the bulk series has values in the range of 45–50 nm, which is larger than the corresponding values of the nano samples. This is an expected result attributable to grain thermal growth during sintering process. The calculated particle size using SEM shown in Fig. 5 was inaccurate. The inaccuracy is due to the clustering of the particles in these sintered materials.

**Figure 6:**
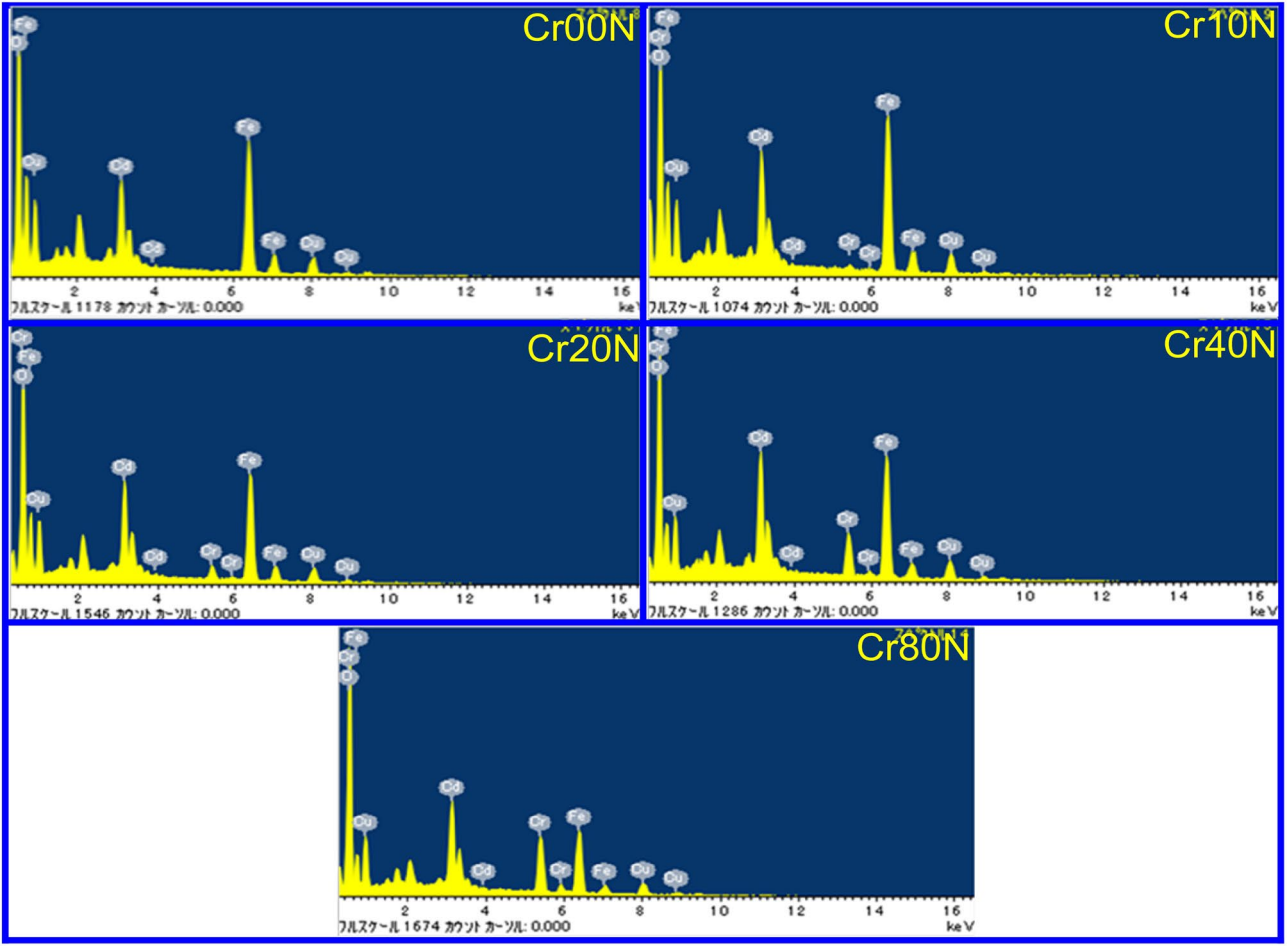
Energy dispersive x-ray spectroscopy of the as-prepared samples.

By EDX, the sample elemental composition and purity were confirmed. Figure 6 shows the EDX spectra of Cd_0.5_Cu_0.5_Cr_x_Fe_2−x_O_4_ consisting peaks of Cd, Cu, Cr, Fe, and O elements and no other peaks present, which confirm that the samples are pure and immune to other impurities^36^. The EDX results proposed the existence of all the elements expected in these spinel ferrite compositions^37^. The result of the EDX analysis shown in Table 6, all EDX observations for divalent (Cd/Cu): trivalent (Cr/Fe): oxygen (O) is close to 1:2:4, which is a perfect match for the stoichiometric and reagent ratios of cations in Cd_0.5_Cu_0.5_Cr_x_Fe_2−x_O_4_^38^.

**Table 6:** The weight percentage (wt%) and the atomic percentage (at.%) of the different ions in the prepared series.

Element	$\mathrm{Cr}00\mathrm{N}$		$\mathrm{Cr}10\mathrm{N}$		$\mathrm{Cr}20\mathrm{N}$		$\mathrm{Cr}40\mathrm{N}$		$\mathrm{Cr}80\mathrm{N}$	
	wt%	at.%	wt%	at.%	wt%	at.%	wt%	at.%	wt%	at.%
$\mathrm{O}$	24.12	56.10	21.80	53.01	24.18	56.69	22.89	54.67	28.44	61.71
$\mathrm{Cr}$	0	0	1.13	0.84	2.90	2.10	8.21	6.04	14.69	9.81
$\mathrm{Fe}$	44.96	29.96	46.13	32.13	40.23	27.03	37.74	25.83	25.50	15.85
$\mathrm{Cu}$	14.54	8.51	12.43	7.61	12.74	7.52	10.99	6.61	12.37	6.76
$\mathrm{Cd}$	16.39	5.43	18.51	6.41	19.94	6.66	20.17	6.86	19.00	5.87

### Transmission electron microscope images

**Figure 7:**
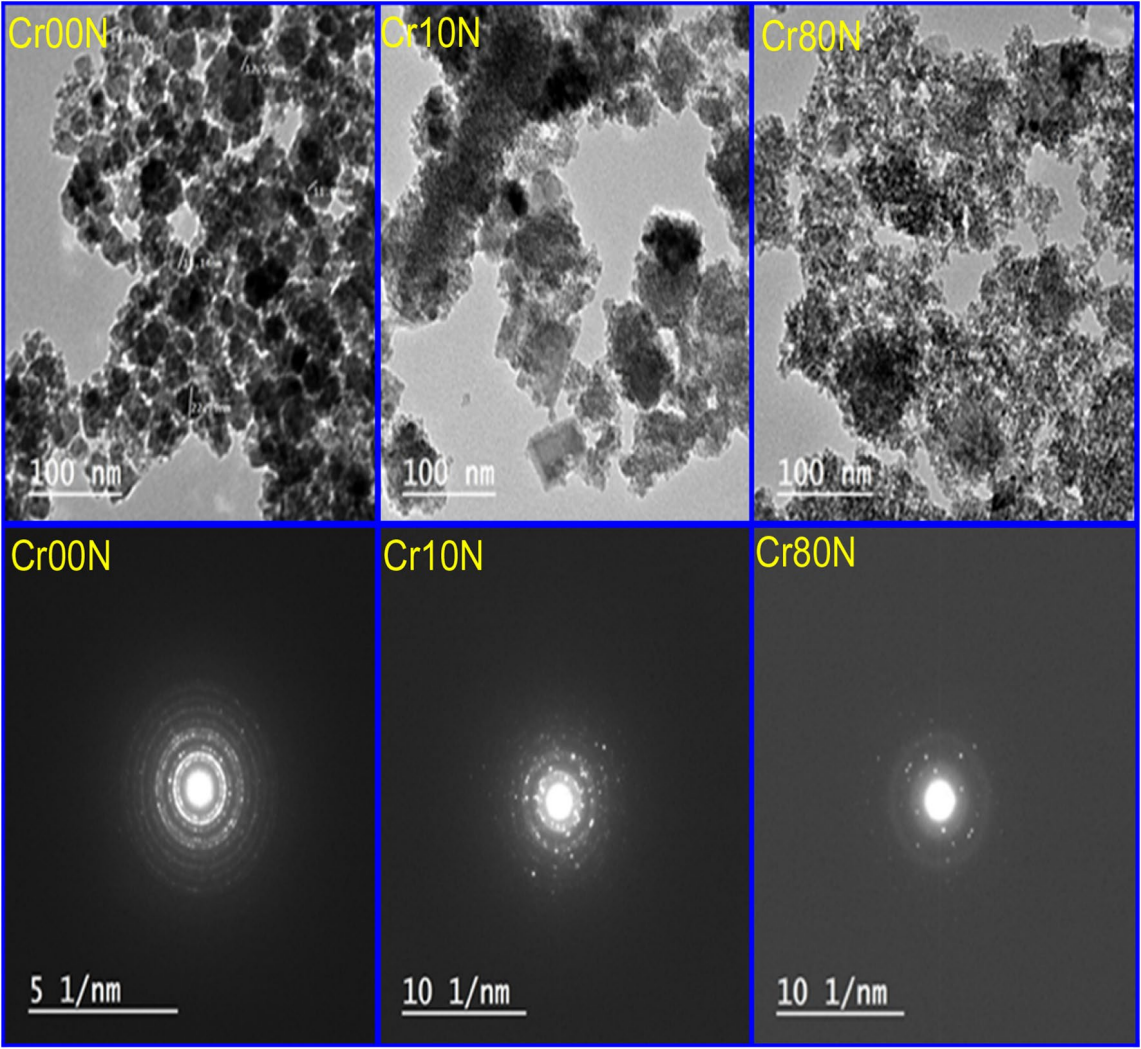
TEM images of as-prepared samples of the Cr00N, Cr10N and Cr80N.

**Figure 8:**
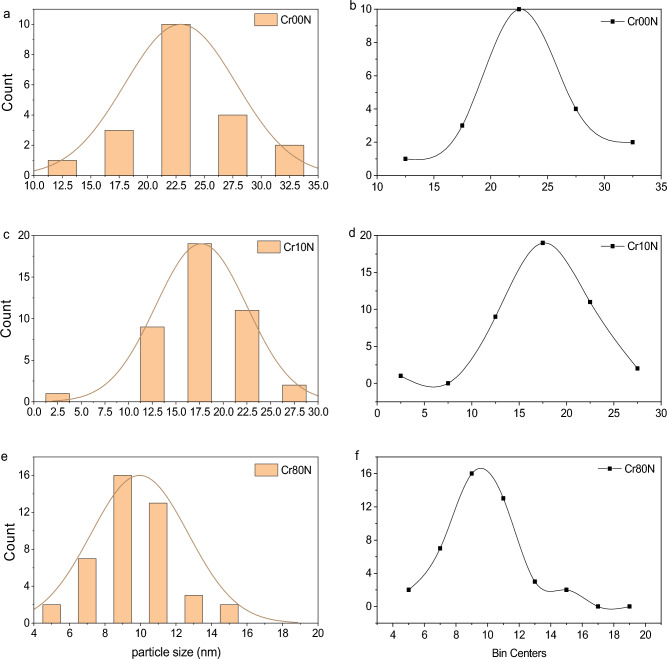
Particle size distribution of as-prepared samples of the Cr00N, Cr10N and Cr80N, from TEM images.

Typical pictures of the as prepared Cd_0.5_Cu_0.5_Cr_x_Fe_2−x_O_4_ nanoparticles are shown in Fig. 7. It is shown that the nanoparticles aggregate, which may indicate that ferromagnetically structured nanoclusters, have formed^39^. The observed average values of particle sizes (R) range from 9 to 22.5 nm, as shown in Fig. 8 and reported in Table 7. This indicates that the grain size is in the same range as the corresponding crystallite size, revealing that each grain consists of approximately one crystallite. The selected area electron diffraction measurements (SAED) show that the material grains crystallize to nanosize.

**Table 7:** The average grain size from TEM images.

Sample	Cr00N	Cr10N	Cr80N
R (nm)	22.5	17.5	9

**Figure 9:**
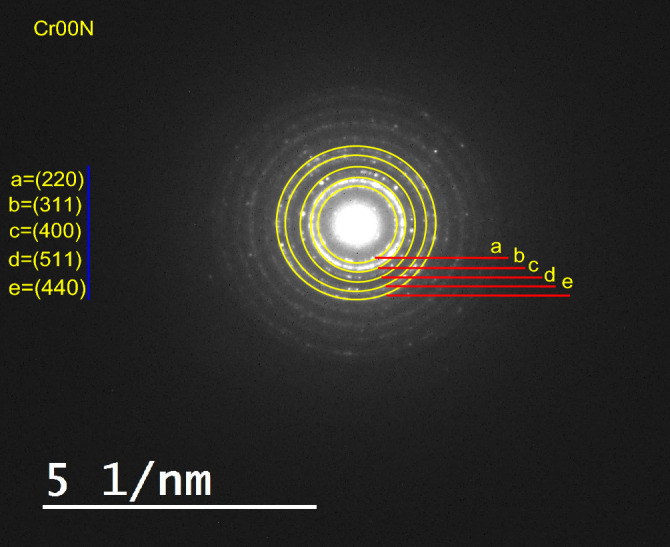
Selective area electron diffraction (SAED) pattern for sample Cr00N.

Figure 9 display the selected area electron diffraction (SAED) pattern consists of sharp concentric rings with sharp bright spots over the rings, which is an indication of the polycrystalline nature^40^. Table 8 display d-space result from SAED, the obtained d-spacing values for Cr00N nano ferrite are correlated with that from XRD results; indicating the presence of different lattice fringes having lattice spacing of 0.3 nm, 0.255 nm, 0.214 nm, 0.162 nm, and 0.149 nm, respectively to (220), (311), (400), (511), and (440) planes. Similar results for SAED of cubic spinel nano ferrites were obtained^37^.

**Table 8:** Calculated d-spacing calculated from Rietveld refinement and SAED correlated to miller indices (hkl) for sample Cr00N.

2Theta	29.68	34.95	42.47	56.14	61.63
d-Space (Rietveld)	3.008	2.565	2.127	1.637	1.504
d-Space (SAED)	3.007	2.55	2.143	1.629	1.497
hkl	220	311	400	511	440

### Raman spectroscopy

**Figure 10:**
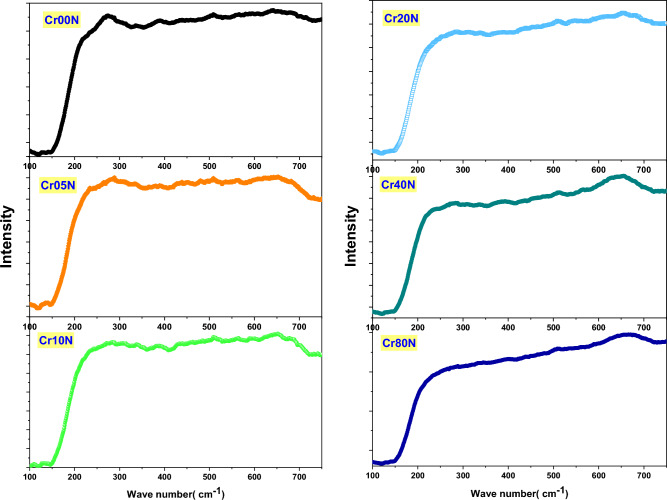
Raman spectra of the as-prepared samples.

Figure 10, shows the general patterns of the Raman active modes for the Cd–Cu nanoferrite samples that have Cr added. The line broadening and peak shifting in Raman peaks corresponded to nanocrystalline ferrites. The shifting line broadening of Raman peaks is caused by the lack of long-range order as well as the effects of surface pressure and phonon confinement, which are usually present in nanometer-sized materials. The nanograins’ Brillouin zone folding, which prevents phonon propagation, activates all of the phonons' Raman modes. The broad Raman line spectra can represent five peaks at 190 cm^−1^, 282 cm^−1^, 300, 480 cm^−1^and 665 cm^−1^. When compared to the pattern of pure Cd–Cu ferrite, the peaks showed a trendy blue shift. The bond length shrank with the replacement of Cr, as evidenced by the blue shift of vibrational modes, which is in good accord with the increased lattice parameter predicted from the XRD study^41,42^.

**Figure 11:**
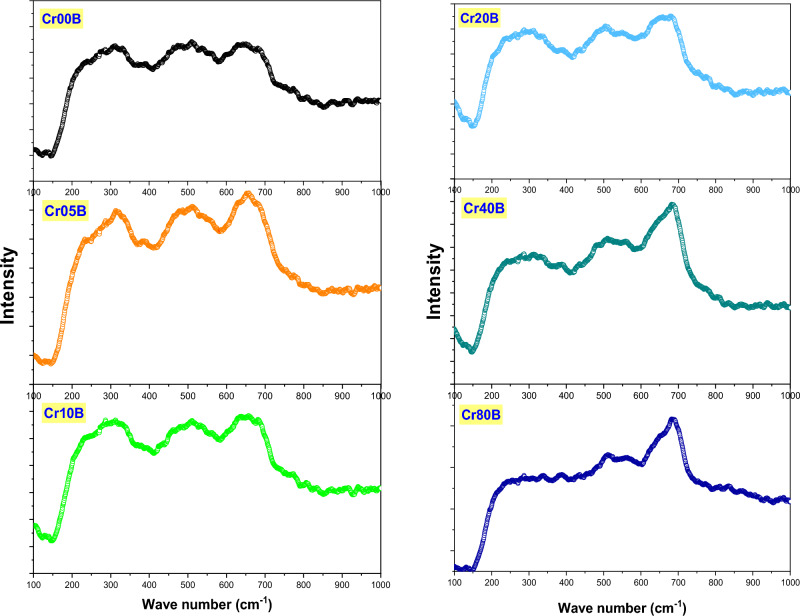
Raman spectra of the bulk samples.

Figure 11, displays the Raman spectra of bulk samples of Cr-doped Cd–Cu ferrite. At lower wavenumbers, the spectra display a broad band; however, at higher wavenumbers, the band tends to become sharper with a smaller shoulder. The broad signals can be deconvoluted into Gaussian-shape signals at five positions: 195, 225, 300, 500, and 665 cm^−1^. According to the group theory of the Raman spectrum for mixed spinel ferrite, as the degree of inversion grows, the expected five internal modes' symmetry is disturbed, and the number of normal modes starts to grow. Accordingly, the shoulders and broadening seen in the signals are immediate results of the non-zero degree of inversion^43^. When the amount of Cr substitution was increased, a strong signal could be seen in the bulk ferrite samples. According to XRD calculations, this signal rapidly increases in synchrony with a lower degree of Cu inversion.

**Figure 12:**
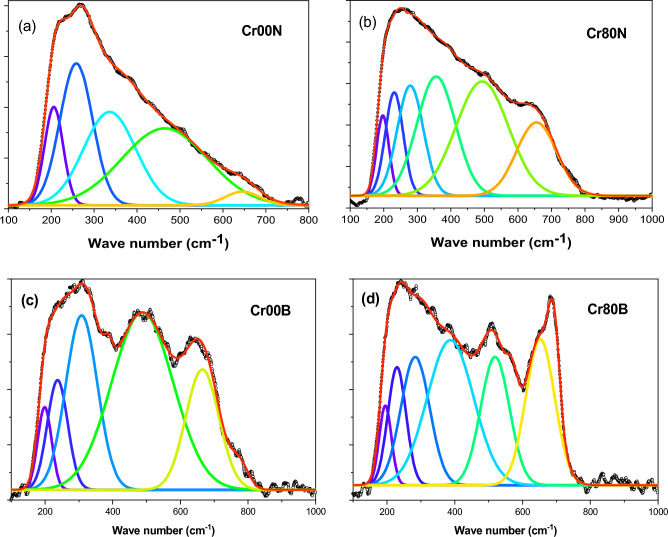
(**a**) and (**b**): two fitted as-prepared samples (x = 0.0 and 0.8), (**c**) and (**d**): Two fitted bulk samples (x = 0.0 and 0.8).

Tables 9 and 11 provide the fitting parameters for the Raman spectra for the two-ferrite series, nanoferrite samples, and bulk samples. The mode references are shown in Table 11. Figure 12a,b represents the fitting of the Raman spectra for the two nano ferrite samples (x = 0.0 and 0.8). The tetrahedral site mode has the highest frequency at 665 cm^-1^. This shows that the local lattice has an effect on the tetrahedral sub-lattice. The peak intensity in this region implies a small change in the cation disorder with increasing Cr concentrations, along with a degree of long-range order^44^. On the other hand, the peak at 480 cm^−1^ has been ascribed to the octahedral site mode, displaying the local lattice influence in the octahedral sub-lattice. Trivalent cation disorder is responsible for the broad peak in this area, which shows a lack of short-range order in the spinel phase (Fe^3+^ and Cr^3+^). The fact that the Raman spectra aren’t symmetrical isn’t because of a bad phase or impurity, because the X-ray diffraction patterns of all of these samples are monophasic. As a result, the asymmetry of Raman peak in this example is caused by disorder in the cation distribution and nanosized particles. The asymmetry is more prominent for nanoparticles of smaller sizes, and it began to decrease as particle size increased. As a result, Raman peak asymmetry is attributed to the presence of cation disorder in smaller particles because of their higher surface-to-volume ratio (spherical particle).

**Table 9:** Represents the fitting parameters modes and FWHM values for the Raman spectra nanoferrite samples.

Sample		Cr00N	Cr05N	Cr10N	Cr20N	Cr40N	Cr80N
T_g_(1)	Xc	–	192	197	194	192	197
	FWHM	–	30	37	34.7	30	36
E_g_	Xc	258	268	277	270	266	279
	FWHM	76	73	76	75	74	77
T_g_(2)	Xc	336	336	348	338	342	356
	FWHM	121	110	113	114	114	114
T_g_(3)	Xc	464	466	479	486	480	493
	FWHM	199	172	173	193	140	156
A_g_	Xc	645	643	650	661	656	655
	FWHM	80	135	135	121	117	118

**Table 10:** Represents the fitting parameters modes and FWHM for the Raman spectra for the bulk samples.

Sample		Cr00B	Cr05B	Cr10B	Cr20B	Cr40B	Cr80B
T_g_(1)	Xc	198	192	191	191	194	194
	FWHM	48.3	31	32	29	35	35
E_g_	Xc	236	227	223	224	230	229
	FWHM	71	55	50	44	57	53
T_g_(2)	Xc	308	308	283	266	297	283
	FWHM	109	98	89	63	93	84
T_g_(3)	Xc	487	491	494	507	512	518
	FWHM	181	154	141	187	89	84
A_g_	Xc	664	661	656	660	670	692
	FWHM	98	95	80	80	89	32

**Table 11:** Provide the mode references.

Formula	A_1g_ cm^-1^	T_2g_(3) cm^-1^	T_2g_(2) cm^1^	T_2g_(1) cm^-1^	E_g_ cm^-1^	Reference
CuFe_2_O_4_	$675 {\mathrm{Fe}}^{3+}-{\mathrm{o}}^{2-}$	550 Fe or Cu	450 Bond Fe–O/Cu–O	**–**	–	^48^
	$$\mathrm{Cu}-\mathrm{o}$$					
CuFe_1.5_Cr_0.5_O_4_	670	560	480	–	–	^48^
CuFeCrO_4_	660	580	520	–	–	^48^
CuFe_1.5_ Cr_0.5_O_4_	685	–	–	–	–	^48^
CuCr_2_O_4_	680 Cr–O	590 Cr–O	530	–	440 Cr–O	^48^
c-CFO	700 Fe^3+^–O^2−^	–	–	–	–	^49^
	656 Cu^2+^–O^2−^					
t-CFO	700 Fe^3+^–O^2−^	–	–	–	–	^49^
	653 Cu^2+^–O^2−^					
Ni_1-x_ Zn_x_Fe_2_O_4_	698	571	477	229	333	^50^
Ni_1-x_Cu_x_Fe_2_O_4_	670 A_1g_ (2)	553	495	–	345	^51^
	703 A_1g_ (1)					
Cu_0.4_Co_0.6_Fe_2_O_4_	683.3	599	464	–	301	^52^
CoCuCr_2_O_4_	684.6	*633*	551	139.91	*483*	^53^

**Figure 13:**
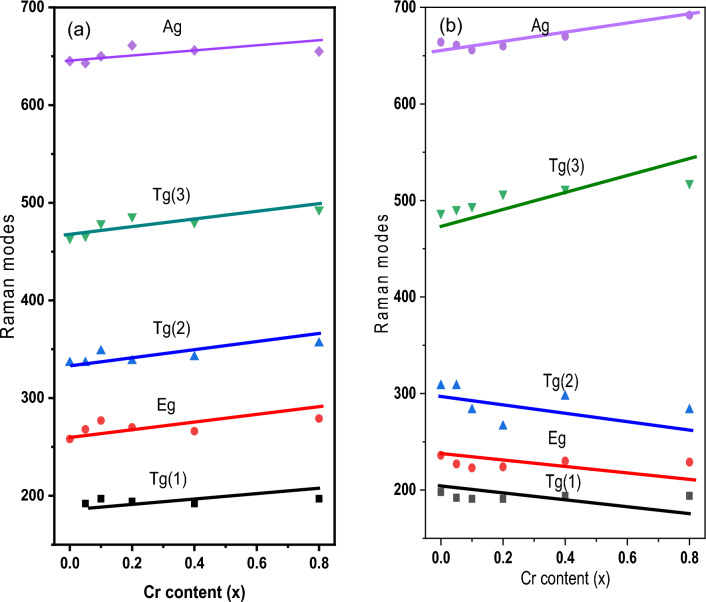
(**a**) the behavior of the Raman modes versus Cr contents of the as-prepared samples and (**b**) the behavior of the Raman modes vs Cr contents of the bulk samples**.**

Figure 12c,d shows how the baseline Raman spectra for two bulk ferrite samples (x = 0 and 0.8) were fit to the three regions. In the first region, from 150 to 400 cm^−1^ as the Cr substitution increased, the band area exhibited asymmetrical changes. Due to a lower degree of Cu inversion, the left lift band shoulder went away, and a higher degree of trivalent cation disorder caused the right lift band to get wider^45^. In the second interval from 400 to 600 cm^−1^, signal positions show a consistent increase while shouldering and broadening decrease. This behavior may be the result of Cr regular substitution, which has a zero-degree inversion from the octahedral to the tetrahedral state while Fe has a high degree of inversion. In the third region, from 600 to 800 cm^−1^, the broadening decreased and the Ag mode was sharp with a small shoulder at the left side of the tetrahedral signal. This showed a significant change in Cu ion transfer from the octahedral site into the tetrahedral site and a lower degree of inversion as the Cr content went up. In Fig. 13a,b, the position of the Raman mode's band is affected by strain and loss of symmetry, which has been reported before^46,47^ and is in line with the presented observations. It is important to note that these two factors have an effect on the position of the Raman mode's band. In the case of mixed ferrite nanocrystals containing two or more divalent cations, induced strain can cause some disorder, but non-stoichiometry and other variables can cause a loss of symmetry, vacancies, lattice defects, or the positioning of metal ions at their locations. Because of the lack of symmetry, modes shift and line widths broaden^45^.

### The electronic absorption- spectroscopy

**Figure 14:**
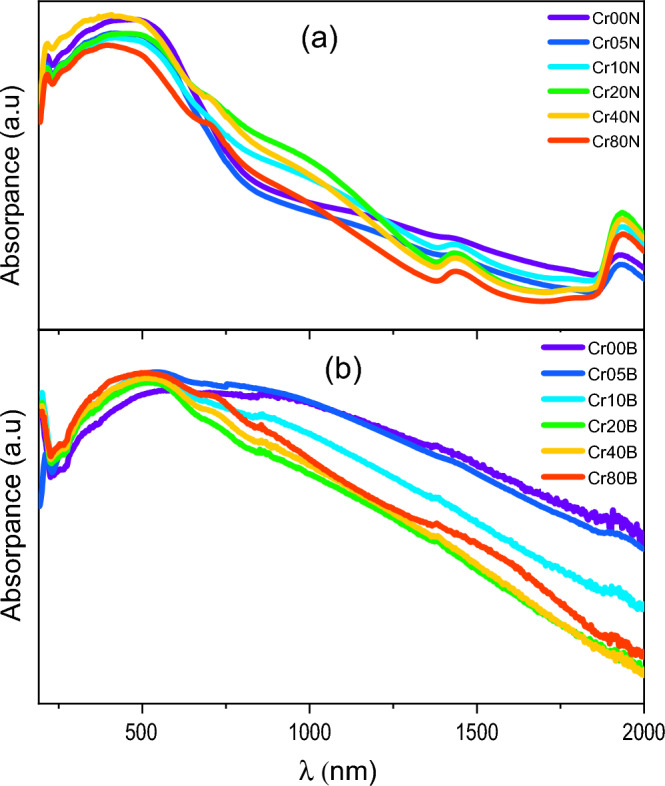
(**a**) UV-visible absorption spectra of as-prepared samples and (**b**) bulk samples.

DRS is used to describe the optical characteristics of the samples. A critical change in reflectance (cut-off frequency) around (λ = 568 nm, photon energy = 2.185 eV) was observed. The DRS of the nanoparticle ferrite sample is shown in Fig. 14a has a lot of surface plasmonic resonance (SPR) when light with a wavelength of 500 nm hits it and makes its free electrons vibrate, causing tearing forces that are stopped by the coulomb force^54–56^. The tuning of surface plasmonic wavelength into near infrared (NIR) at 1500 and 2000 nm may be attributed to the dispersed size and corner-sharpness of nanostructures of nanostructure ferrite, especially with increasing chromium ion doping. Figure 14b is the DRS of bulk ferrite samples. The surface plasmonic resonance band gets wider in the visible region, and the two bands in the NIR disappear when the nanostructures and sharp corners of the particles are destroyed by heating, which causes the particles to stick together.

The analysis of optical absorption spectra can help to understand the band gap and band structure of nanoferrite particles. The optical band-gap values were found by using the Kubelka–Munk (K-M) function, F(R), in Eq. (11). This function is found by figuring out the sample's diffuse reflectance^35^. The K-M formula relates diffuse reflectance R to absorption amount K and scattering quantity S:

11 $$\mathbf{F}\left(\mathbf{R}\right)=\frac{\mathbf{K}}{\mathbf{S}}={\left(1-\mathbf{R}\right)}^{2}/2\mathbf{R}$$

**Figure 15:**
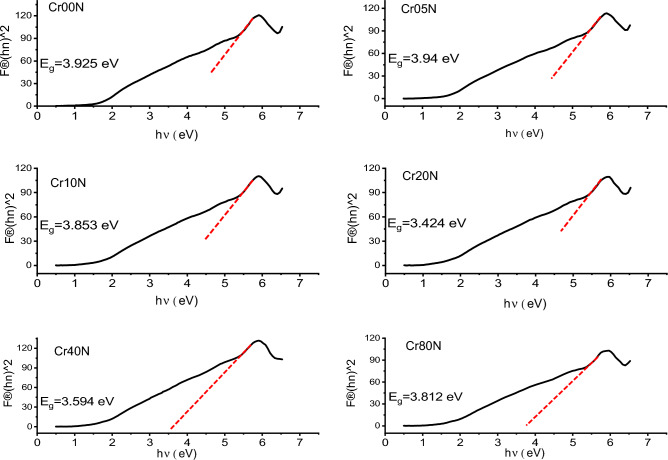
Band gap Tauc plot of the as-prepared samples.

**Figure 16:**
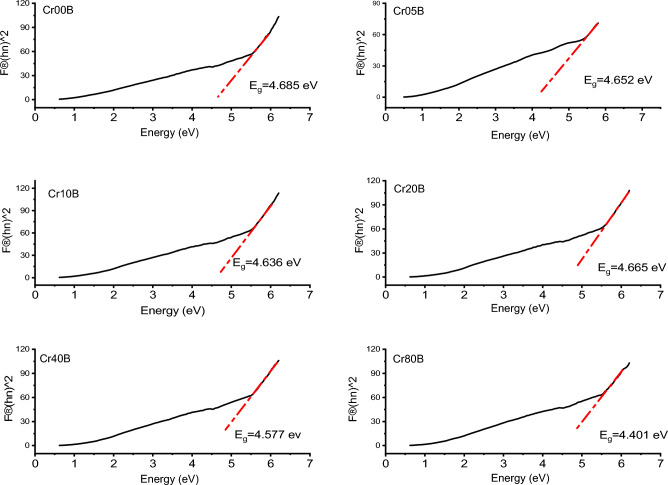
Band gap Tauc`s plot of the bulk samples.

Assuming that S doesn’t change much over the wavelength range of electronic absorption, the band gap was found using Tauc’s plots (Figs. 15 and 16) and the Kubelka–Munk reemission function, as follows:

12 $$\mathbf{F}\left(\mathbf{R}\right)\mathbf{h}{\varvec{\upnu}}=\mathbf{A}{\left(\mathbf{h}{\varvec{\upnu}}-{\mathbf{E}}_{\mathbf{g}}\right)}^{\mathbf{n}}$$

where $\mathbf{h}{\varvec{\upnu}}$ is the incident photon's energy, A is a constant, and n is an index that depends on the type of transition allowed (n = 1/2). Table 12, shows the energy band-gap **E**_**g**_ determined using Tauc’s plot^57^. The plots of ${[\mathbf{F}(\mathbf{R})\mathbf{h}{\varvec{\upnu}}]}^{2}$ against $\mathbf{h}{\varvec{\upnu}}$ indicate that the optical band-gap is produced by extrapolating the linear part of ${[\mathbf{F}(\mathbf{R})\mathbf{h}{\varvec{\upnu}}]}^{2}$ to the **hν**-axis at ${[\mathbf{F}(\mathbf{R})\mathbf{h}{\varvec{\upnu}}]}^{2}$ = 0. As shown in Table 12, the straight-line plots in Fig. 15 show that the band gap of Cr-doped Cadmium copper nano-ferrite samples is between 3.59 and 3.925 eV. Table 13 shows that as the concentration of Cr^3+^ go up in bulk ferrite samples, the energy band gap goes from 4.085 to 4.65 eV. The energy band gap increases as particle size decreases. This can be explained using the Bras effective mass model^58^.

**Table 12:** Represents the energy gap ${\mathrm{E}}_{\mathrm{g}}$, E_0_, E_d_, ${{\varvec{\uplambda}}}_{0}$ and peak frequency ${{\varvec{\uplambda}}}_{\mathbf{p}\mathbf{e}\mathbf{a}\mathbf{k}}$ of the as prepared samples.

Sample	${\mathrm{E}}_{\mathrm{g}}$	E_0_	E_d_	${\uplambda }_{0}$	${\uplambda }_{\mathrm{peak}}$
	(eV)	(eV)	(eV)	(nm)	(nm)
Cr00N	3.925	1.357	− 3.400	911.81	470
Cr05N	3.94	1.305	− 4.585	949.53	424
Cr10N	3.853	1.102	− 5.845	1126.85	422
Cr20N	3.424	1.119	− 4.104	1114.0	450
Cr40N	3.594	1.265	− 2.966	984.94	428
Cr80N	3.812	1.211	− 4.911	1032.2	394

**Table 13:** Represents the energy gap ${\mathrm{E}}_{\mathrm{g}}$, E_0_, E_d_, ${{\varvec{\uplambda}}}_{0}$ and peak frequency ${{\varvec{\uplambda}}}_{\mathbf{p}\mathbf{e}\mathbf{a}\mathbf{k}}$ of the bulk samples.

Sample	${\mathrm{E}}_{\mathrm{g}}$	${\mathrm{E}}_{0}$	**E**_**d**_	${\uplambda }_{0}$	${\uplambda }_{\mathrm{peak}}$
	(eV)	(eV)	(eV)	(nm)	(nm)
Cr00B	4.685	4.364	4.935	911.81	567
Cr05B	4.652	3.452	1.598	949.53	542
Cr10B	4.636	3.780	2.235	1126.85	521
Cr20B	4.665	4.585	4.309	1114.03	524
Cr40B	4.577	4.532	3.703	984.94	516
Cr80B	4.401	4.445	2.804	1032.24	507

The band gap decreases with an increase in Cr^3+^ content in nanoferrite samples and bulk samples. Crystallite size, structural characteristics, and the presence of defects are just a few of the variables that affect the values. In this sequence, the decrease in E_g_ with Cr addition can be attributed to two factors: (i) lowering the lattice parameter. (ii) the occurrence of localized electronic states in the specimen^29^. The smaller band gap may also be caused by the sp-d exchange interaction between the localized d-electrons of Cr^3+^ ions and the band electrons of Cd_0.5_Cu_0.5_Fe_2_O_4_. So, the fact that Cr^3+^ doping made the band gap smaller could be due to the formation of subbands between the energy band gaps, which then merged with the conduction band to make a single band^57^. In nano- and bulk-ferrite samples, the band gap energy decreases as the crystallite size decreases. Both the band gap energy and the crystallite size have the same trend as Cr content increases.

**Figure 17:**
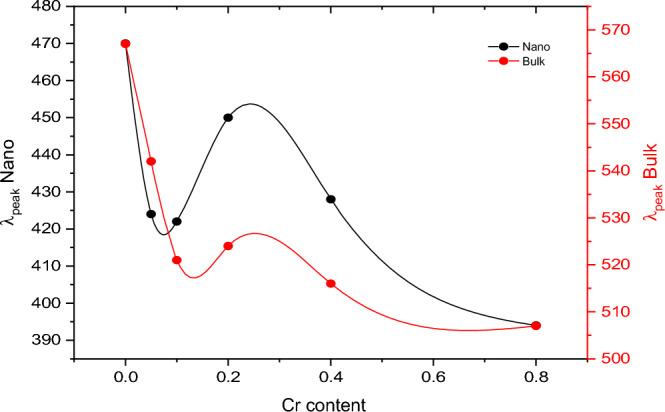
The variation of cut off frequency with increasing the doping content in as-prepared and bulk ferrites.

Based on what we’ve talked about so far, the energy band gap of the Cr-doped ferrite nanoparticles that were made shows how dopants change the basic lattice structure ^9^. The presence of Cr, which introduces many charge carriers that induce an increase in ${\mathbf{E}}_{\mathbf{g}}$ values, is the main factor in increasing ${\mathbf{E}}_{\mathbf{g}}$ values. The increase in band gap caused by adding Cr ions leads the samples to become insulators due to the increased distance between the valance and conduction bands. The size reduction seen with dopant concentration in crystallite size measurements supports the theory that the quantum confinement effect is responsible for this increase^59^. Figure 17 shows the cutoff frequency for Cd–Cu–Cr ferrites in the range (550 nm) for bulk ferrites and (420 nm) for nano ferrites, as well as the cutoff frequency in the visible region, so that these materials can be used in non-linear optical devices. industrial catalysts, semiconductors, solar energy conversion devices, etc^5^.

The index of refraction, or N, of a material is an important feature; it is a complex quantity made up of real and imaginary terms. The real term, n, relates to the real index of refraction, which indicates the amount by which an electromagnetic wave slows in comparison to its velocity in a vacuum. The extinction coefficient, or extinction index, k, is an imaginary number that quantifies the amount of an electromagnetic wave that has been absorbed and scattered inside the material. The index of refraction is given by^60^:

13 $$\mathrm{N}(\uplambda ) =\mathrm{ n}(\uplambda ) +\mathrm{ i k}(\uplambda )$$

The extinction coefficient k was determined in this paper using the following equation^60^:

14 $$\mathrm{k}=\mathrm{\lambda \alpha }(\uplambda )/2\uppi ={\left(1-\mathrm{R}\right)}^{2}$$

where **λ** is the wavelength of the incident light and **α(λ)** is the absorption coefficient. The extinction coefficient k denotes the amount of incident light dissipated per unit length of material due to scattering and absorption processes. The refractive index n is computed as a function of λ, R and k using the equation

15 $$\mathrm{n }=\frac{1 +\mathrm{ R}}{1 -\mathrm{ R}}+{\left[\frac{4\mathrm{R}}{{\left(1-\mathrm{R}\right)}^{2}}-{\mathrm{K}}^{2}\right]}^\frac{1}{2}$$

The refractive indices of various materials for various photon energies can be calculated using a variety of simplified models. The semi-empirical relationship known as "The Wemple-DiDominico Dispersion Relation" is one of these models. which measures the intensity of optical transitions between bands. The following Eqs. (16), (17) show how the dispersion energy, which is given by the (${\mathbf{E}}_{\mathbf{d}}$) parameter, and the excitation energy needed for electronic transitions, which is given by the single oscillator energy ${(\mathbf{E}}_{0})$, are both related to the refractive index, n:^61,62^:

16 $${\mathrm{n}}^{2}=1+\frac{{\mathrm{E}}_{0}{\mathrm{E}}_{\mathrm{d}}}{{{\mathrm{E}}_{0}}^{2}-{(\mathrm{hv})}^{2}}$$

17 $$\frac{1}{{\mathbf{n}}^{2}-1}=\frac{{\mathbf{E}}_{0}}{{\mathbf{E}}_{\mathbf{d}}}-\frac{{(\mathbf{h}\mathbf{v})}^{2}}{{\mathbf{E}}_{0}{\mathbf{E}}_{\mathbf{d}}}$$

**Figure 18:**
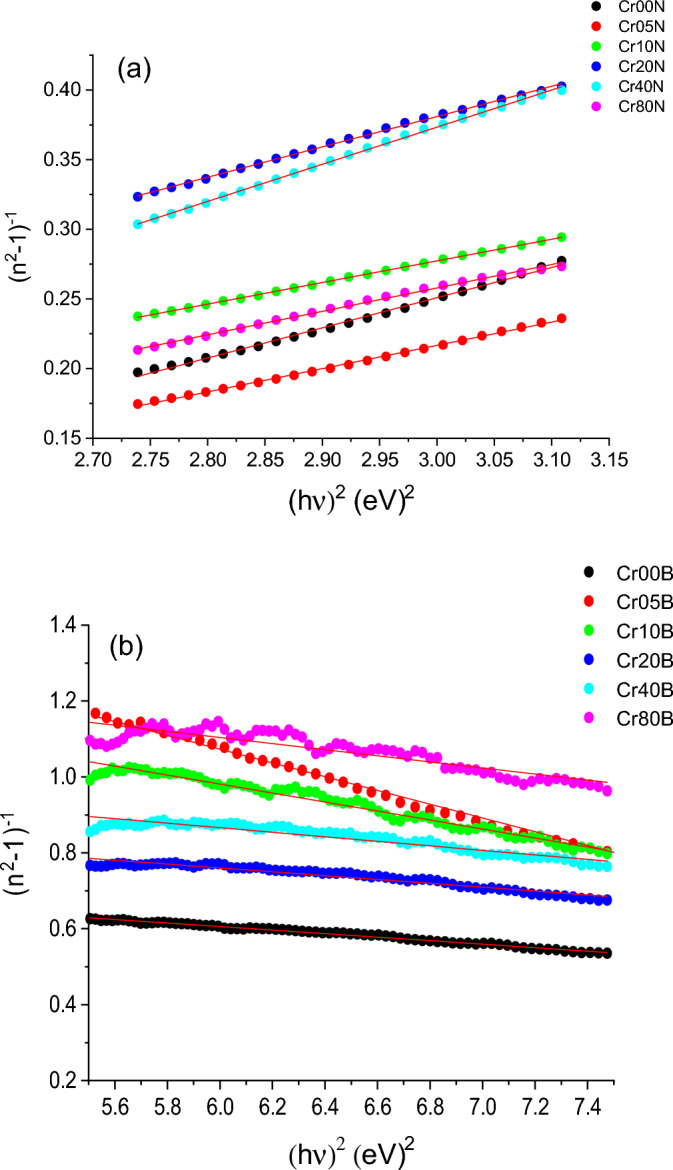
(**a**) and (**b**): Plot of (n^2^−1)^−1^ versus hυ^2^ for as-prepared and bulk samples respectively.

**E**_**d**_ denotes the strength of optical transitions between bands, which is proportional to the number of free electrons present in the valence band involved in a given transition. **E**_**d**_ is independent of **E**_**g**_ values, but **E**_**0**_, which describes the optical properties of the material, is comparable to **E**_**g**_. Equation (17) and Fig. 18a,b show that the slope and interception point of the lines describe the relationship between ${({\mathbf{n}}^{2}-1)}^{-1}$ and ${(\mathbf{h}\mathbf{v})}^{2}$. Tables 12 and 13 show the values of ${\mathbf{E}}_{\mathbf{d}}$ and ${\mathbf{E}}_{0}$. The ${\mathbf{E}}_{0}$ values increased from 4.079 eV (for x = 0.1) to a higher value of 5.407 eV (for x = 0.8). The minor changes in ${\mathbf{E}}_{0}$ values are expected to be accompanied by minor changes in ${\mathbf{E}}_{\mathbf{g}}$ values due to changes in the inner structure of the band gap caused by Cr doping.

### PL spectroscopy

**Figure 19:**
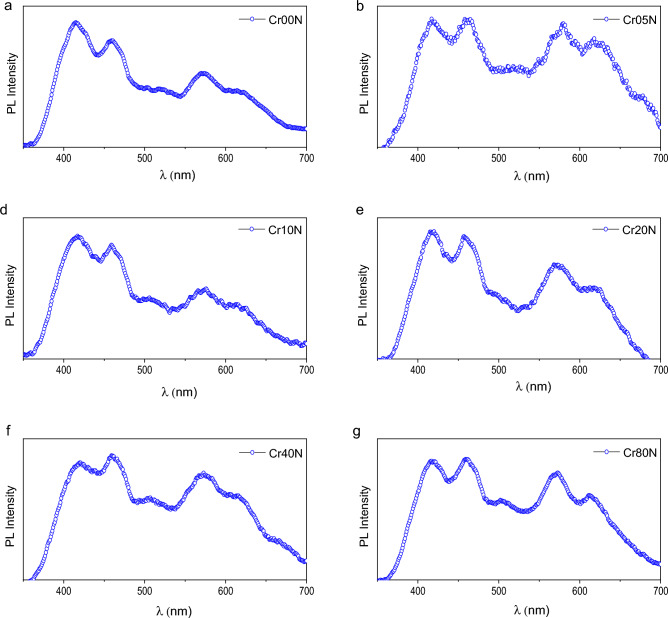
Photoluminescence emission of the as-prepared samples at excitation wavelength 300 nm.

**Figure 20:**
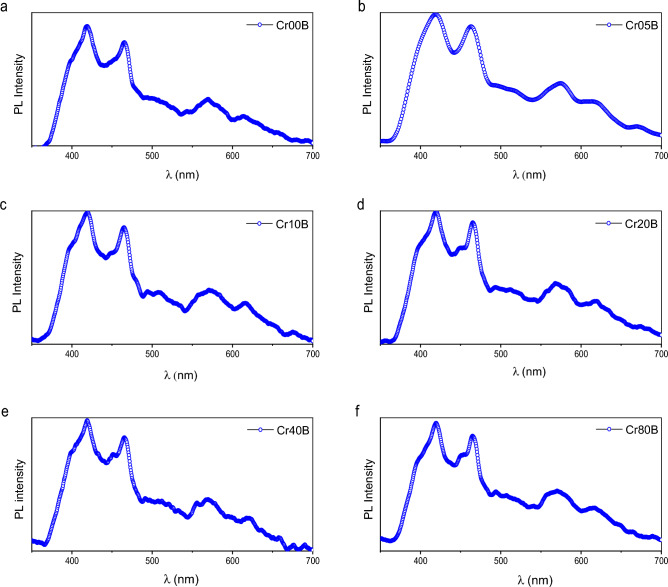
Photoluminescence emission of the bulk samples at excitation wavelength 300nm.

Room-temperature photoluminescence (PL) spectroscopy is often used to study the electrical structure, optical properties, recombination phenomena, and relative energy position of sub-band-gap defect states on metal oxide surfaces^9^. The photoluminescence mechanism is affected by particle size, surface imperfections, and functional groups acquired during the synthesis process^9^. Peaks in the 420–460 nm region were detected as a result of stimulated electron and hole recombination. There were oxygen vacancies and grain boundaries in Cd_0.5_Cu_0.5_Cr_x_Fe_2−x_O_4_ nanoparticles, which caused them to give off blue light between 400 and 500 nm^57^. The photoluminescence spectra of Cd_0.5_Cu_0.5_Cr_x_Fe_2−x_O_4_ particles recorded at room temperature with an excitation wavelength of 300 nm are shown in Figs. 19 and 20.

On excitation with a wavelength of 300 nm, the pattern reveals four emission peaks at 420 nm, 460 nm, 571 nm, and 617.5 nm. When the excitation spectra at 350 nm are changed, the intensity of the PL spectra decreases while the PL peak placements remain the same. The strongest peak is at 420 nm, which is typical of near-band-edge (NBE) blue emission. This emission is most likely made by direct recombination of photo-generated holes formed in the tetrahedral and octahedral sites of the crystals and oxygen vacancy-trapped electrons^40^. Whereas the peak at 460 nm may be attributed to the transitions of Fe^+3^ ions from the 3d^5^ state to the 3d^4^ 4s state, the excited conduction band electron from the localised 3d^5^ state of Fe^3+^ balances the 4s orbital of Fe^3+^^40^. The peaks at 460 nm correspond to blue emission caused by radiative defects caused by point defects at grain boundaries^57^. Yellow emissions at roughly 571 nm are attributed to 3d^5^.

When Cr^+3^ ions replace Fe^3+^ ions in Cd_0.5_Cu_0.5_Fe_2_O_4_ (x = 0.0–0.8), the PL peaks get stronger because of oxygen vacancies and interstitial defects. This is because the distance between the dopant (activator) and the array goes up. The spinel ferrite shows a strong band of luminescence located at 460 nm (2.8 eV); this luminescence can be observed even with the naked eye at room temperature and is due to exciton emission^19^. Calcination of spinel ferrite increases the PL intensity due to an increase in the population of carriers. Applications of photoluminescence (PL) spectroscopy to characterize solid surfaces in terms of adsorption, catalysis, and photocatalysis, where PL spectra can tell important information about the local structure of catalytically active sites and their photocatalytic activities^63^, On the other hand, the band gap energies decreased with increasing Cr^3+^ content, and due to the larger surface area of the investigated ferrite due to its small crystallite size, the probability of photocatalytic activity increased^64^. The broad peak found for nano samples at 420nm and 460nm is attributed to violet emission attributed to Cr^+3^ vacancies in the lattice, which vanishes after calcination in the bulk sample, as shown in Fig. 19^65^. Furthermore, the two primary PL peaks were identified at around 420 and 460 nm, which are typically caused by oxygen vacancies and interstitial defects^35^. S. Yuvaraj et al.^66^ reported similar results, with two primary PL peaks seen at 420 and 466 nm. However, it was shown that increasing the concentration of Cr^+3^ ions (x = 0.0–0.8) in Cd_0.5_Cu_0.5_Fe_2_O_4_ matrices improved the PL intensity due to an increase in the distance between the dopant (activator) and the array^57^.

A big part of the emission process is played by the defect centers that act as trap levels and by the role of Cr^+3^ activators in making the PL intensity of Cr^+3^-doped materials stronger. The abnormal augmentation of PL intensity during the sintering process can be explained by an increase in the population of carriers. The extra carriers are provided by surface states with directionally localized exactions^67^. The introduction of donors and acceptors into the system causes a shift in the PL violet emission peak from 415 to 420 nm by raising the Cr^+3^ content^9^. The surface shape and condition of the produced nanoparticles are verified to be sensitive and have a substantial influence on the determination of PL spectra because particle size is affected by heat treatment.

**Figure 21:**
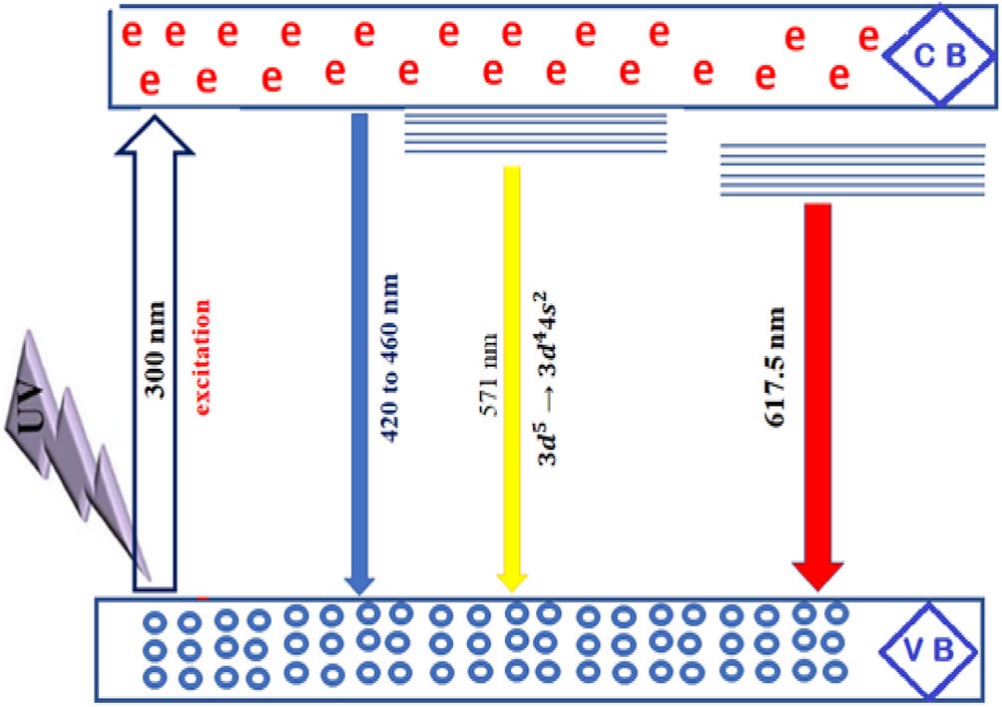
Scheme of excitation and photoluminescence emission of difference route of deactivation.

The presence of more Cr dopants in bulk samples reduces free carrier mobility. As they draw closer to the charged dots, the free carriers scatter. A decrease in mobility diminishes their capacity to recombine by increasing the carrier separation, and hence the emission of photoluminescence decreases. We think that when the amount of Cr doping at ferrite goes down, more radiative recombination centers are made and the intensity of photoluminescence emission goes up^68^. Figure 21 is a sketch of an energy level diagram that shows the full method of PL emission by doping spinel ferrite with Cr^+3^. The excited electrons can unite with holes in the ground state via numerous mechanisms, including radiative or non-radiative recombination. The blue band showed up between 420 and 460 nm because oxygen-vacancy-trapped electrons combined directly with photo-generated holes in the tetrahedral and octahedral sites of the crystals^40^. Yellow emissions seen at 571 nm are ascribed to the ${\text{3d}}^{{5}} \, \to \,{\text{3d}}^{{4}} {\text{4s}}^{{2}}$ transition of (Fe^+3^) ions. As illustrated in Fig. 21, the radiative shift from interstitial Cr^+3^ states to the valence band yields red emission^69^. By calcination, the intensity of the yellow–red emission peak reduces and virtually vanishes with higher concentrations of Cr^+3^.

### Relation between UV–Visible and PL spectra (Stokes shift)

**Figure 22:**
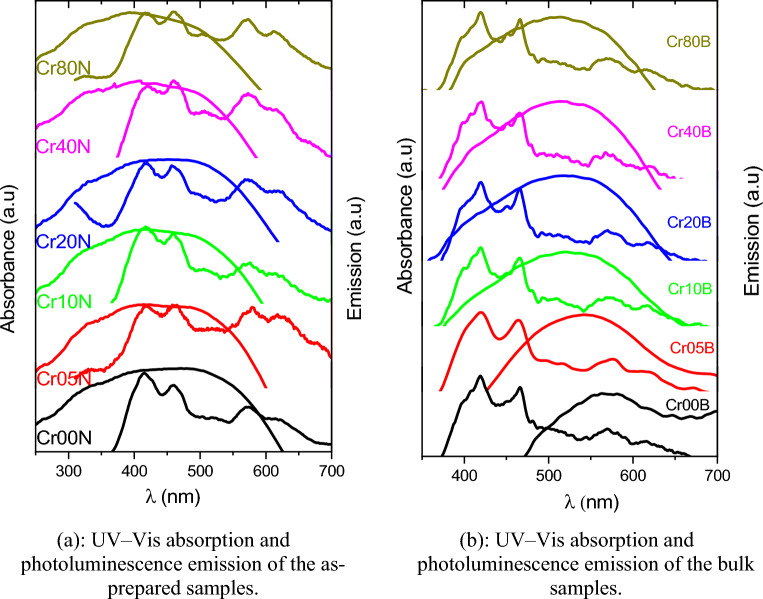
(**a**): UV-Vis absorption and photoluminescence emission of the as-prepared samples. (**b**): UV-Vis absorption and photoluminescence emission of the bulk samples.

Figure 22a,b represents the difference between the maximum absorbance and emission wavelength of various emission peaks. This difference may be attributed to a different pathway of the deactivation process; the decreasing stock shift of PL_4_ may be due to an increasing Cr^3+^ ratio with respect to Cu^2+^ in octahedral, which caused a decrease in the repulsion in energy level where Cu contains a d^9^ electron, so with increasing Cr^+3^, the emitted gap energy increases, which may be confirmed by calculated gap energy as in Tables 14 and 15.

**Table 14:** Energy gaps and photoluminescence parameters for as-prepared samples.

Sample	${\lambda }_{\mathrm{PL}1}$	${\lambda }_{\mathrm{PL}2}$	${\lambda }_{\mathrm{PL}3}$	${\lambda }_{\mathrm{PL}4}$	Stoke shift 1	Stoke shift 2	Stoke shift 3	Stoke shift 4	${\mathrm{E}}_{\mathrm{gPL}1}$	${\mathrm{E}}_{\mathrm{gPL}2}$	${\mathrm{E}}_{\mathrm{gPL}3}$	${\mathrm{E}}_{\mathrm{gPL}4}$
	(nm)				${\mathrm{cm}}^{-1}$				$(\mathrm{eV})$			
Cr00N	415	459	570	614	9236	11,546	15,789	17,046	2.991	2.704	2.178	2.022
Cr05N	417	458	580	618	9352	11,499	16,091	17,152	2.977	2.710	2.140	2.008
Cr10N	417	460	576	614	9352	11,594	15,972	17,046	2.977	2.698	2.155	2.022
Cr20N	420	457	568	619	9523	11,451	15,727	17,178	2.955	2.716	2.185	2.005
Cr40N	420	459	573	–	9523	11,546	15,881	–	2.955	2.704	2.166	–
Cr80N	417	459	573	611	9352	11,546	15,881	16,966	2.977	2.704	2.166	2.032

**Table 15:** Energy gaps and photoluminescence parameters for bulk samples.

Sample	${\lambda }_{\mathrm{PL}1}$	${\lambda }_{\mathrm{PL}2}$	${\lambda }_{\mathrm{PL}3}$	${\lambda }_{\mathrm{PL}4}$	Stoke shift 1	Stoke shift 2	Stoke shift 3	Stoke shift 4	${\mathrm{E}}_{\mathrm{gPL}1}$	${\mathrm{E}}_{\mathrm{gPL}2}$	${\mathrm{E}}_{\mathrm{gPL}3}$	${\mathrm{E}}_{\mathrm{gPL}4}$
	(nm)				$({\mathrm{cm}}^{-1})$				(eV)			
Cr00B	419	465.5	570	614	9467	11,851	15,789	17,047	2.962	2.666	2.178	2.022
Cr05B	419	463	575	620	9467	11,735	15,942	17,201	2.962	2.681	2.159	2.002
Cr10B	420	465	571	616	9524	11,828	15,820	17,100	2.955	2.669	2.174	2.015
Cr20B	419.5	465	568	617	9495	11,828	15,728	17,126	2.959	2.669	2.185	2.012
Cr40B	419	466	570	618	9467	11,851	15,789	17,152	2.962	2.666	2.178	2.008
Cr80B	419.5	465	570	617	9495	11,828	15,789	17,126	2.959	2.669	2.178	2.012

## Conclusion

Using the co-precipitation method, six nanoferrite samples of Cd–Cu ferrites with Cr added have been made successfully. The six prepared samples were sintered at 1000 °C for 2 h. The cubic spinel ferrite structure is corroborated by XRD patterns for both the six as-prepared and the six thermally treated samples. The as-prepared samples have crystallite sizes ranging from 10 to 20 nm, whereas the sintered samples have crystallite sizes ranging from 40 to 50 nm, highlighting the significance of the sintering process on grain growth. By increasing the chromium content, the lattice parameters are reduced. SEM images illustrate how agglomeration intensifies as doping ion concentrations rise.

For Cr-doped Cd–Cu nanocrystalline ferrites, the Raman active mode patterns showed that the lines got wider and the peaks moved. Comparing the Raman line spectra to the pattern of a pure Cd–Cu nanoferrite sample, the peaks showed a trendy blue shift in good accord with the decreased lattice parameter calculated from the XRD study. Raman spectra of Cr-doped Cd–Cu bulk samples displayed a broad band at lower wavenumbers and a sharper band with a smaller shoulder at higher wavenumbers, revealing a non-zero degree of Cu inversion. When the amount of Cr substitution was increased, a stronger signal could be seen in the bulk ferrite Raman spectra than in the corresponding nanoparticle Raman spectra. The asymmetrical feature of Raman spectra is not a result of an undesirable phase or impurity; the asymmetry is due to disorder in cation distribution and nanosized particles.

The band gap energies resulting from Tauc`s plot decreased with increasing Cr^3+^ content. PL A spectral emission shift was expected to occur upon particle growth by calcination. The violet emission peak at 420cm^−1^ was shifted to a higher wavelength. The wavelengths of photoluminescence emission and UV-Vis absorption are closely coincident.

## Data Availability

The data that support the findings of this study are available from the corresponding authors, [R. E. El shatter, and A. W. Awad], upon reasonable request.
